# Structure and specificity of the RNA-guided endonuclease Cas9 during DNA interrogation, target binding and cleavage

**DOI:** 10.1093/nar/gkv892

**Published:** 2015-10-10

**Authors:** Eric A. Josephs, D. Dewran Kocak, Christopher J. Fitzgibbon, Joshua McMenemy, Charles A. Gersbach, Piotr E. Marszalek

**Affiliations:** 1 Department of Mechanical Engineering and Materials Science, Edmund T. Pratt, Jr. School of Engineering, Duke University, Durham, NC 27708, USA; 2 Department of Biomedical Engineering, Edmund T. Pratt, Jr. School of Engineering, Duke University, Durham, NC 27708, USA; 3 Center for Genomic and Computational Biology, Duke University, Durham, NC 27708, USA; 4 Department of Orthopaedic Surgery, Duke University Medical Center, Durham, NC 27710, USA

## Abstract

CRISPR-associated endonuclease Cas9 cuts DNA at variable target sites designated by a Cas9-bound RNA molecule. Cas9's ability to be directed by single ‘guide RNA’ molecules to target nearly any sequence has been recently exploited for a number of emerging biological and medical applications. Therefore, understanding the nature of Cas9's off-target activity is of paramount importance for its practical use. Using atomic force microscopy (AFM), we directly resolve individual Cas9 and nuclease-inactive dCas9 proteins as they bind along engineered DNA substrates. High-resolution imaging allows us to determine their relative propensities to bind with different guide RNA variants to targeted or off-target sequences. Mapping the structural properties of Cas9 and dCas9 to their respective binding sites reveals a progressive conformational transformation at DNA sites with increasing sequence similarity to its target. With kinetic Monte Carlo (KMC) simulations, these results provide evidence of a ‘conformational gating’ mechanism driven by the interactions between the guide RNA and the 14th–17th nucleotide region of the targeted DNA, the stabilities of which we find correlate significantly with reported off-target cleavage rates. KMC simulations also reveal potential methodologies to engineer guide RNA sequences with improved specificity by considering the invasion of guide RNAs into targeted DNA duplex.

## INTRODUCTION

**Figure 1.**
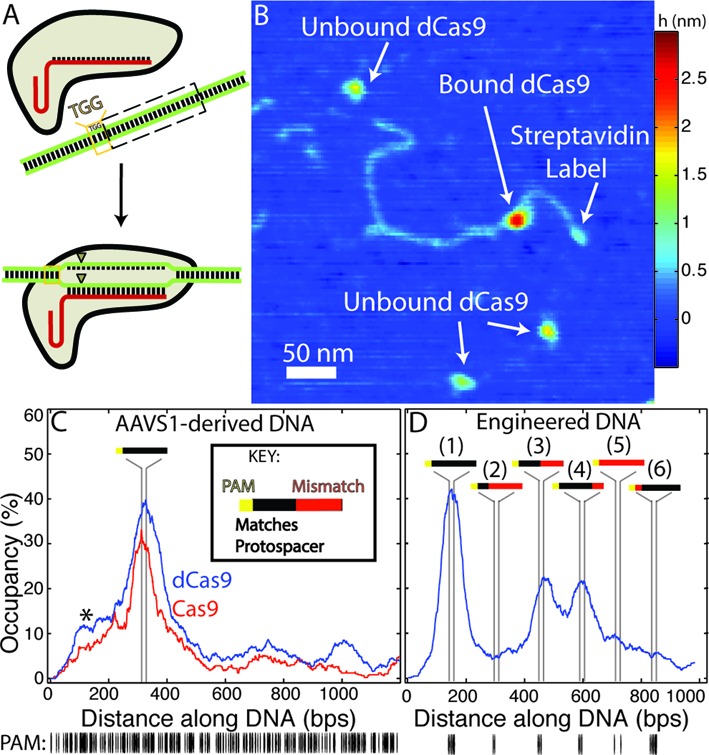
(**A**) Schematic representation of Cas9 activity: having bound a single-guide RNA (sgRNA, red), the Cas9-sgRNA complex binds to 20 bp ‘protospacer’ sequences in a targeted DNA molecule, provided that the protospacer is directly followed by a protospacer adjacent motif (PAM, here ‘TGG’). Following binding, the Cas9 endonuclease produces double-strand breaks (triangles) within the protospacer. (**B**) Atomic force microscopy (AFM) image of dCas9-sgRNA bound at the protospacer sequence within a single streptavidin-labelled DNA molecule derived from the human AAVS1 locus. (**C**–**D**) Fraction of bound DNA occupied by Cas9/dCas9-sgRNA along an AAVS1-derived (**C**) or an engineered DNA substrate (**D**) designed with a series of fully-complementary and partially-complementary protospacer sequences. Vertical lines represent the (23 bp) segments where each significant feature is located on the respective substrates (see inset key). (**C**) dCas9 and Cas9 exhibit nearly identical binding distributions on the AAVS1 substrate (*n* = 404 and *n* = 250, respectively). The asterisk marks an off-target ‘shoulder peak’ in the binding distribution (see text). (**D**) On the engineered substrate (*n* = 536) dCas9 binds with the highest propensity to the complete protospacer with no mismatched (MM) sites (peak 1, later referred to as the full or ‘0MM’ site) and also to sites with 10 or 5 mismatched bases distal to the PAM site (third and fourth feature from streptavidin label, referred to later as the ‘10MM’ or ‘5MM’ sites, respectively) albeit with the reduced affinity. Sites containing greater numbers of mismatches (second and fifth feature), or which possess two PAM-proximal mismatched nucleotides (sixth feature) are bound at significantly lower rates. (below) Distribution of PAM (‘NGG’) sites in each substrate.

Cas9 is the endonuclease of the prokaryotic type II CRISPR (clustered, regularly interspaced, short palindromic repeats)—CRISPR-associated (Cas) response to invasive foreign DNA (1,2). During this response, Cas9 is first bound by a CRISPR RNA (crRNA) : *trans*-activating crRNA (tracrRNA) duplex, and then directed to cleave DNA that contain 20 base-pair (bp) targeted sites known as ‘protospacers’ that are complementary to a variable 20 nucleotide (nt) segment of the crRNA (Figure 1A). Essentially, the only constraint on sequences that Cas9 can target is that a short protospacer adjacent motif (PAM), such as ‘NGG’ in the case of *S. pyogenes* Cas9 (1), must immediately follow the protospacer sites in the foreign DNA molecule. Cas9's ability to be modularly ‘programmed’ by bound RNA molecules to target nearly any DNA site has recently generated tremendous excitement after CRISPR-Cas9 systems were re-appropriated for a number of heterologous biotechnological applications (3,4). Notably, a single-guide RNA (sgRNA) hairpin has been designed which combine the essential components of the crRNA : tracrRNA duplex into a single functional molecule (1,5). With this sgRNA, Cas9 can be introduced into a variety of organisms to produce targeted double strand breaks *in vivo* for remarkably facile genomic engineering (4,6,7). Nuclease-null Cas9 (D10A/H840A, known as ‘dCas9’) and chimeric dCas9 derivatives have also been used to alter gene expression *via* targeted binding at or near promotor sites *in vivo* (8–11) as well as to introduce targeted epigenetic modifications (12). However, there have been several reports of off-target binding and cleavage by Cas9 (13–17), which can adversely affect its potential uses in practice. Significant efforts have been made to characterize this off-target activity—and to improve specificity of Cas9/dCas9 through intelligent selection of protospacer target sequences (18–20); optimization of sgRNA structure, *e.g*. by truncation of first two 5′- nucleotides in the sgRNA (21); and use of ‘dual-nicking’ Cas9 enzymes (22). A clear understanding of the precise mechanism of RNA-guided cleavage as it relates to the structural biology of Cas9 will be essential to developing Cas9 derivatives and guide RNAs with increased fidelity for their emerging applications in medicine and biology.

Pursuant to this goal, here we use AFM to resolve individual *S. pyogenes* Cas9 and dCas9 proteins as they bind to targets along engineered DNA substrates after incubation with different sgRNA variants. This technique allows us to directly resolve both the binding site and structure of individual Cas9/dCas9 proteins simultaneously, providing a wealth of mechanistic information regarding Cas9/dCas9 specificity with single-molecule resolution. Consistent with traditional biochemical studies, we find that significant binding by Cas9/dCas9 with sgRNAs occurs at sites containing up to 10 mismatched base-pairs in the target sequence. However, while use of guide RNAs with two nucleotides truncated from their 5′- end (tru-gRNA) had previously shown to result in up to 5000-fold decrease in off-target mutagenesis by Cas9 *in vivo*, we find similar specificities *in vitro* for dCas9 with tru-gRNA binding to mismatched targets as with standard sgRNA. The addition of a hairpin to the 5′- end of the sgRNA which partially overlaps the target-binding region of the guide RNA is found to increase dCas9 specificity at the cost of decreased overall binding propensity to DNA. Our results indicate that overall stability of guide RNA-DNA binding does not necessarily govern specificity in Cas9 cleavage when mismatches are located more than 10 bp away from the PAM.

Furthermore, with AFM we are able to directly map the structures of individual Cas9 and dCas9 molecules to the protospacer or off-target sites to which they are bound. We identify a distinct and progressive structural transformation in Cas9 and dCas9 as they bind to mismatched target sites that are increasingly complementary to the protospacer sequence, which provides evidence of a ‘conformational gating’ mechanism that regulates RNA-guided specificity. In combination with kinetic Monte Carlo (KMC) experiments, these results suggest that this observed conformational shift in the structural properties is stabilized by interactions between the guide RNA and the protospacer at or near the 14th–17th nucleotide positions from the PAM. Furthermore, we find that the stability of the guide RNA at these positions correlates with reported rates of off-target cleavage significantly better than guide RNA–protospacer binding energy alone. These results implicate differential sources of mismatch tolerance or sensitivity by Cas9 according to their guide RNA structures and suggest new avenues to design guide RNAs with improved specificities.

## MATERIALS AND METHODS

### Materials

Tris-HCl (pH 7.6) buffer was obtained from Corning Life Sciences. L-glutamic acid monopotassium salt monohydrate, dithiothreitol (DTT) and magnesium chloride were obtained from Sigma Aldrich Co., LLC.

### Cloning of Cas9, dCas9 and sgRNA expression plasmids; expression and purification of Cas9, dCas9; expression and purification of sgRNA and guide RNA variants; and generation of DNA substrates

Plasmids encoding Cas9, dCas9 and sgRNAs which target the AAVS1 locus of human chromosome 19 were cloned, expressed and purified using standard techniques (23). The DNA substrates used for imaging—(i) a 1198 bp substrate derived from a segment of the AAVS1 locus of human chromosome 19; (ii) an ‘engineered’ 989 bp DNA substrate containing a series of six full, partial or mismatched target sites; and (iii) a 1078 bp ‘nonsense’ substrate containing no homology to the protospacer (>3 bp)—were also generated using standard techniques. See Supplementary Methods for details. Sodium dodecyl sulfate-polyacrylamide gels of purified Cas9 and dCas9 are presented in Supplementary Figure S1 which indicate they are approximately 95% pure.

### Atomic force microscopy (AFM)

Atomic force microscopy (AFM) was performed in air using a Bruker (née Veeco) Nanoscope V Multimode with RTSEP (Bruker) probes (nominal spring constant 40 N/m, resonance frequency, 300 kHz). Prior to experiments, protein and guide RNAs were mixed in 1:1.5 ratio for 10 min. Protein and DNA were mixed in a solution of working buffer for at least 10 min (up to 35 min) at room temperature, deposited for 8 s on freshly cleaved mica (Ted Pella, Inc.) that had been treated with 3-aminopropylsiloxane (prepared as previously described (24)), rinsed with ultra-pure (>17 MΩ) water, and dried in air. Proteins were centrifuged briefly prior to incubation with DNA. When the standard sgRNA was used, at least four preparations for each experimental condition were imaged, and at least two preparations imaged for experiments with the other guide RNA variants. In general images were acquired with pixel resolution of 1024 × 1024 over 2.75 micron square areas or 2048 × 2048 over 5.5 micron square areas at 1–1.5 lines/s for each sample. Images of several thousand (∼2500–6000) DNA molecules were resolved for each experimental condition.

### DNA tracing and refinement with sub-pixel resolution

Acquired AFM images were flattened and leveled (plane-wise, by line and by third order polynomial leveling) using an open-source image analysis software for scanning probe microscopy, Gwyddion (http://gwyddion.net/), and then exported to MATLAB (Mathworks, Inc.). 151 × 151 pixel (405 nm × 405 nm) regions containing each DNA molecule were sorted by inspection for a clearly identifiable streptavidin label, the presence of at least one bound Cas9/dCas9 molecule, and an unambiguous end-to-end path to ensure lack of aggregation or overlap with other DNA molecules. The contour of the DNA was traced by hand and the estimated boundaries of the streptavidin and Cas9/dCas9 were marked. The trace was then algorithmically refined using a method inspired by Wiggins *et al*. (25). See Supplementary Methods for details of the algorithmic refinement.

**Figure 2.**
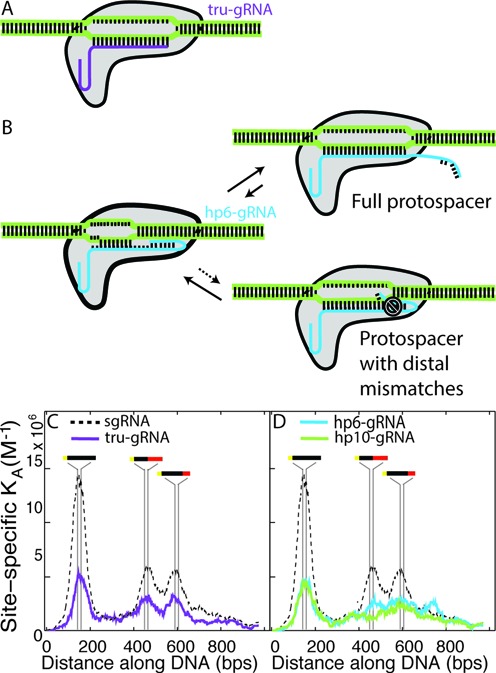
Modulation of binding affinity and specificity by guide RNA variants. (**A**) Schematic of dCas9 bound to a single-guide RNA with a two nucleotide truncation from its 5′- end (tru-gRNA, purple). (**B**) Schematic and proposed mechanism of dCas9 bound to a single-guide RNA with 5′- end extension that forms a hairpin with the PAM-distal binding segment of its targeting region (hp-gRNA, blue). After a PAM site is bound and strand invasion of the DNA by the guide RNA has initiated, the hairpin is opened upon binding to a full protospacer and full strand invasion can occur. If there are PAM-distal mismatches at the target site, then it is more energetically favourable for the hairpin to remain closed and strand invasion is hindered. (**C**) Single-site binding affinities (K_A_) for dCas9 with tru-gRNA (purple, *n* = 257) along the engineered DNA substrate (see Figure 1D). Dashed line shows the site-specific affinities of dCas9-sgRNA for comparison. The binding distribution of dCas9 with tru-gRNAs exhibits distinct peaks in its affinity exactly at the protospacer sites with 10 PAM-distal mismatches and 5 PAM-distal mismatches, demonstrating that it does not have increased binding specificity relative to full sgRNAs (see Table 1). (**D**) Single-site binding affinities (K_A_) for dCas9 with guide RNAs with 5′- hairpins that overlap the nucleotides complementary to the last 6 (hp6-gRNA, blue) or 10 (hp10-gRNA, green) PAM-distal nucleotides of the protospacer. The specific peaks at the sites with 5 and 10 distal mismatches are significantly flattened, with dCas9 and hp10-gRNA exhibiting substantially decreased affinity for off-target sites (22% drop relative to dCas9 with tru-gRNA in their maximum observed off-target binding affinity, with a decrease of up to 57% at sites with 10 PAM-distal mismatches). The peaks in affinity at the full protospacer sites imply that the hairpins indeed open upon full invasion. *n* = 243 for hp6-gRNA and *n* = 212 for hp10-gRNA.

The binding histograms of Figures 1C–D and 2C–D, and Supplementary Figure S2 were generated by mapping the relative location of each bound protein to the bases overlapped (nearest-neighbour interpolation) by the protein and summing the total number of proteins bound to each site (if a single Cas9/dCas9 could be interpreted as being in contact with multiple (*k*) sites, each region of contact was weighed by 1/*k* in the binding histogram). Peaks in the binding histogram were fit to the empirical Gaussian exp(−((x−μ)/w)^2^), where μ is the mean peak position and w is the peak width parameter (w = √2σ, with σ the standard deviation), using MATLAB.

### Determination of dCas9 apparent dissociation constants

Apparent dissociation constants of dCas9 with different guide RNA variants were determined using the method pioneered by Yang *et al*. (26). Briefly, at known solution concentrations of dCas9-guide RNA ([dCas9]_0_) and DNA molecules ([DNA]_0_), we count the respective numbers of ‘engineered’ DNA molecules with and without proteins bound (fraction of DNA bound by proteins *Θ*_dCas9_), and after tracing DNA with bound proteins (see above) we determine the average number of proteins bound per DNA molecule (*n*_dCas9_). Overall dissociation constants are calculated as

\begin{equation*} \begin{array}{*{20}l} {K_{{\rm d},{\rm DNA}} = [{\rm DNA}][{\rm dCas}9]/[{\rm DNA} \bullet {\rm dCas}9] = } \\ {(1 - \Theta _{{\rm dCas}9} )([{\rm dCas}9]_0 - n_{{\rm dCas}9} [{\rm DNA}]_0 )/(\Theta _{{\rm dCas}9} )} \\ \end{array} \end{equation*}

The protospacer-specific dissociation constants *K*_d,protospacer_ are calculated similarly using instead *Θ*_dCas9,protospacer_, the fractions of DNA with dCas9 bound within one peak width of the Gaussian fit in their respective binding histograms (*i.e*. see Table 1), as are the site-specific association constants *K*_a,ss_ = *K*_d,ss_^−1^ using the fractions of each site on the DNA with a bound dCas9 *Θ*_dCas9,ss_.

**Table 1.** Peaks recorded in binding histograms of Figure 1C–D for Cas9/dCas9-sgRNA and Figure 2C for dCas9 with sgRNAs possessing 2 nt truncation at 5′- end (tru-gRNA), based on empirical fit to Gaussian ∝ exp(−((x−μ)/w)^2^)

Guide RNA:	sgRNA^a^			tru-gRNA^b^			sgRNA	
Substrate:	Engineered DNA:			Engineered DNA:			AAVs1-derived DNA:	
Total DNA molecules traced:^c^	*n* = 536			*n* = 257			*n* = 404	*n* = 250
Location name:	Full site	10 MM site^d^	5 MM site^e^	Full site	10 MM site^f^	5 MM site^g^	Full site	Full site
Cas9/dCas9	dCas9	dCas9	dCas9	dCas9	dCas9	dCas9	dCas9	Cas9
Location:^h^	144–167	452–465	592–610	144–167	452–465	592–610	316–339	316–339
Peak μ^i^ (95% conf.):	151.3 (151.1, 151.6)	467.6 (466.6, 468.5)	600.6 (599.5, 601.7)	159.0 (158.2, 159.7)	462.9 (462.1, 463.6)	592.0 (590.9, 593.0)	327.7 (327.3, 328.2)	315.0 (314.4, 315.7)
Peak width^j^ w = √2σ (95% conf.):	51.46 (51.53, 52.38)	57.5 (55.84, 59.16)	70.8 (68.72, 72.89)	53.98 (52.2, 55.76)	54.44 (52.07, 56.81)	67.88 (64.27, 71.49)	84.10 (83.12, 85.27)	58.7 (56.8, 60.63)
# dCas9^k^:	287	180.5	211.9	84.5	58.75	74.33		
# / (2w) (scaled to density at full site, 95% conf.):	1	0.5688	0.5399	1	0.6894	0.6994		

^a^Standard single-guide RNA (sgRNA).

^b^Single-guide RNA with 2 nt truncated from 5′- end (tru-gRNA).

^c^Numbers of DNA molecules observed with both monovalent streptavidin label and bound protein which were then traced (see Supporting Methods for details).

^d^Target site with 10 PAM-distal mismatched nucleotides.

^e^Targeted site with 5 PAM-distal mismatched nucleotides.

^f^On the engineered DNA substrate, tru-gRNA is expected to interact with only the first 8 of the 10 PAM-distal mismatched nucleotides at the 10 MM site.

^g^On the engineered DNA substrate, tru-gRNA is expected to interact with only the first 3 of the 5 PAM-distal mismatched nucleotides at the 5 MM site.

^h^bp from streptavidin-labelled end (from PAM to end of site).

^i^Peak maximum in binding histogram (from Gaussian fit).

^j^Peak width is √2σ, with σ as the standard deviation.

^k^Number of dCas9 molecules observed within 1 peak width (√2σ) of binding site. If Cas9/dCas9 appeared to contact DNA at n sites, that molecule is weighted by 1/n. If molecules overlapped both 10 MM and 5 MM sites, # was weighted by an additional 1/2.

### Protein alignment and clustering

Images of Cas9 and dCas9 proteins which were isolated and appeared only to contact the DNA at a single location were extracted. These features were selected as those with features greater than (μ_d_ + 2σ_d_) which fit entirely within a 134 nm × 134 nm bounding box, where μ_d_ and σ_d_ are the mean and standard deviation of the DNA height to which the proteins are bound; this step essentially had the effect of removing most of the aggregated/densely packed Cas9/dCas9 from the set as well as those proteins from images with larger extrinsic noise. After 4-fold nearest-neighbour interpolation, features of the protein with topographical height greater than (μ_d_ + σ_d_) were each aligned by repeated translation, rotation, and reflection with respect to one another to minimize the mean-squared difference between their topographical heights. A distance matrix was composed of these minimized mean-square differences, then the proteins with standard sgRNA were clustered according to this criterion using the method of Rodriguez and Laio (27); proteins with the guide RNA variants were clustered according to the closest Cas9/dCas9 structure with the standard sgRNA. Ensemble average structures were extracted by performing a reference-free alignment across each member of individual clusters following the method of Penczek, Radermacher and Frank (28). Properties of Cas9/dCas9 populations at each feature (such as protospacer sites) on the DNA were determined using proteins bound within one peak width of the Gaussian distributions fit to the binding histograms (*i.e*. see Table 1).

### KMC of guide RNA strand invasion and R-loop ‘breathing’

KMC experiments to simulate strand invasion by the guide RNAs at protospacer sites were performed using a Gillespie-type (continuous time, discrete state) (29) algorithm implemented in MATLAB. Strand invasion is modeled as a one-dimensional random walk in a position-dependent potential determined by the relative nearest-neighbour dependent DNA:DNA and RNA:DNA binding free energies. See, *e.g*. Figure 4A, and the Supplementary Methods for details. Free energy parameters are derived from the literature for experiments performed at 1 M NaCl at 37°C. Sequence-dependent DNA:DNA hybridization free energies ΔG°(x)_DNA:DNA_ were obtained from (30); sequence-dependent RNA:DNA hybridization free energies ΔG°(x)_RNA:DNA_ were obtained from (31); and ΔG°(x)_RNA:DNA_ values in cases of introduced point mismatches rG·dG, rC·dC, rA·dA and rU·dT were obtained from (32) (under slightly higher salt conditions).

### Correlations between R-loop stability derived from KMC and experimental Cas9 cleavage rates

The sequences of guide RNAs and targeted DNA from Hsu *et al*. (18) with single-nucleotide PAM-distal (≥10 bp away from the PAM site) mismatches of type rG·dG, rC·dC, rA·dA and rU·dT and the experimentally determined maximum likelihood estimate (MLE) cutting frequencies by Cas9 at those sites were imported (*n* = 136) into the KMC script. Simulations of strand invasion initiated at *m* = 10 were repeated 1000 times for each sequence to obtain the mean fraction of time *m* ≥ 16 and correlated with the empirical cleavage rates. Significance was determined by bootstrapping the mean fraction of occupancy with the MLE cutting frequencies *via* permutation 100 000 times, then recalculating correlation coefficients and *P*-values. Guide RNA–protospacer binding free energies were estimated by summing over the nearest-neighbour energies using the parameter sets listed above and corrected with a −3.1 kcal mol^−1^ initiation factor (30).

## RESULTS AND DISCUSSION

### Atomic force microscopy captures Cas9/dCas9 binding specifically and non-specifically along engineered DNA substrates with high resolution

The analysis of crystallographic and biochemical experiments suggests that specificity in protospacer binding and cleavage is imparted first through the recognition of PAM sites by Cas9 itself (33). This event is followed by strand invasion of the DNA by the bound RNA complex and direct Watson-Crick base-pairing between the guide RNA and the protospacer (34) (Figure 1A). However, a complete mechanistic picture of the discrimination or tolerance by Cas9 (prior to cleavage) of DNA sequences that are similar or partially homologous to the protospacer has yet to emerge. To directly probe the relative propensities to bind to protospacer and off-target sites with single-molecule resolution, 50 nM Cas9-sgRNA or dCas9-sgRNA complexes targeting the AAVS1 locus of human chromosome 19 were imaged by AFM in air after incubation with one of three DNA substrates (2.5 nM):

- a 1198 bp segment of the AAVS1 locus containing the complete target site following a PAM (here ‘TGG’) (Figure 1C);
- a 989 bp engineered DNA substrate containing a series of six complete, partial or mismatched target sites each separated by approximately 150 bp (Figure 1D). Mismatches at these sites could span both the ‘seed’ (PAM-proximal, approximately 12 bp) and ‘non-seed’ (PAM-distal) regions of the protospacer. The only PAM sites in this engineered substrate were at these explicitly designed locations; and
- a 1078 bp ‘nonsense’ DNA substrate with 161 PAM sites but no homology (beyond 3 bp sequences) with the target sequence (Supplementary Figure S2).

Structurally, *S. pyogenes* Cas9 is a 160 kDa monomeric protein approximately 10 nm × 10 nm × 5 nm (from crystal structures) (35,36), roughly divided into two lobe-like halves each containing a nuclease domain. Consistent with the X-ray structures, dCas9–sgRNA imaged *via* AFM appears as large ovular structures (Supplementary Figure S3), and after incubating Cas9 or dCas9 with DNA we observe these structures bound along DNA which we assign to be Cas9 or dCas9, respectively (Figure 1B, Supplementary Figures S3 and S4). To unambiguously determine the orientation of the DNA and the sequence of the sites bound by Cas9 and dCas9, the biotinylated DNA molecules were labelled at one end with monovalent streptavidin tag (37) prior to AFM imaging. DNA molecules that were observed with bound Cas9 or dCas9 proteins were selected for further analysis and traced with sub-pixel resolution according to a modified protocol adapted from that of Wiggins *et al*. (25), and the sites bound by Cas9/dCas9 were extracted (see Supplementary Methods for details).

This method proved remarkably robust (Table 1): on the DNA bound by Cas9 or dCas9, a distinct enrichment of proteins centred precisely at the location of protospacer sites with an adjoining PAM (within the expected 23 bp, Figure 1C–D) is observed and manifest as sharp peaks. No such obvious peaks are observed in the DNA substrate containing no target sites (Supplementary Figure S2). Standard deviation of the peak widths ranged from 36–60 bp, which is a significant improvement compared with binding experiments using single-molecule fluorescence that result within peak width standard deviations σ of approximately 1000 bp (34). The mean apparent Cas9/dCas9 ‘footprints’ on DNA cover 78.1 bp ± 37.9 bp; this broadening of the apparent footprint compared to the ∼20 bp footprint of Cas9 on DNA determined by biochemical (35) and crystallographic methods (35,36) is a well-established result of imaging convolution with the width of the AFM tip. Previously, it had been observed *in vitro* that Cas9 remains bound to targeted DNA for extended periods (>10 min) after putative DNA cleavage as a single-turnover endonuclease (34), and could not be displaced from the cleaved strands without harsh chemical treatment. Here too most of the DNA molecules we observed with bound Cas9 appear as full-length AAVS1-derived substrates, with only a small (∼5%) percentage of substrates that have been both cleaved and separated. After these DNA molecules were traced, Cas9 was observed to bind to these ‘full-length’ substrates with nearly an identical distribution as was dCas9 (two-sided Komolgorov-Smirnov test, significance level 5%) (Figure 1C).

By examining the occupancies of dCas9 bound to different locations along the engineered substrate, we can determine the relative binding propensities of dCas9 to various mismatched and partial target sites (Figure 1D, Table 1). The overall dissociation constant between dCas9 and the entire DNA substrate was estimated to be 2.70 nM (±1.58 nM, 95% confidence, Table 2). The dCas9 dissociation constant specifically at the site of the full, or perfectly-matched, protospacer (within one peak width in the binding histogram) located substrate to be 44.67 nM (±1.04 nM, 95% confidence). Earlier electrophoretic mobility shift assays (EMSA) had estimated dCas9-sgRNA binding to protospacer sites on short DNA molecules (∼50 bp) to be between 0.5 nM and 2 nM (34,38). While the increase in dissociation constant at protospacer sites that we observe may be related the presence of multiple off-target sites on the engineered DNA substrate, it is typical that dissociation constants determined by AFM are nearly an order of magnitude higher than those determined by traditional assays (26). This difference is often attributed to non-specific interactions of proteins to the blunt ends of the shorter DNA that are not accounted for in EMSA experiments.

**Table 2.** Apparent dissociation constants for dCas9 with different guide RNA variants from the 989 bp ‘engineered’ DNA substrates (*e.g*. Figures 1D, 2C and D) that contain a series of fully- and partially- complementary protospacer sites

Guide RNA variant	Overall dissociation constant between dCas9 and the engineered DNA substrate (±95% confidence)	Protospacer-specific dissociation constant for dCas9 and the full target on the engineered substrate (±95% confidence)
sgRNA^a^	2.70 nM (±1.58 nM)	44.67 nM (±1.04 nM)
tru-gRNA^b^	17.89 nM (±0.45 nM)	136.4 nM (±2.30 nM)
hp6-gRNA^c^	16.61 nM (±0.40 nM)	164.4 nM (±13.63 nM)
hp10-gRNA^d^	35.84 nM (±0.63 nM)	164.8 nM (±15.60 nM)

^a^Full-length single-guide RNA (sgRNA).

^b^Truncated sgRNA (first two nt at 5′- truncated).

^c^sgRNA with additional 5′- hairpin which overlaps six PAM-distal targeting nts (see text).

^d^sgRNA with additional 5′- hairpin which overlaps ten PAM-distal targeting nts (see text).

On the engineered substrate, dCas9 is relatively tolerant to distal mismatches (exhibiting 50–60% binding propensity relative to complete target site, Figure 1D and Table 1), and has the same apparent affinity (within confidence) towards target sites containing 5 and 10 distal mismatches (MMs). However, binding to protospacer sites containing only two PAM-adjacent mismatches occurred with similar propensity as to sites with 15 or even 20 (PAM site alone) distal mismatches (approximately 5–10% binding propensity relative to perfect target, approximately that of the background binding signal), a finding consistent with previous biochemical studies (34). While there are no PAM sites on the engineered substrate except adjacent to the protospacer sites, on the AAVS1-derived substrate there is a distinct ‘shoulder peak’ of enhanced Cas9 and dCas9 binding near the AAVS1 target that is particularly enriched in PAM sites (Figure 1C, asterisk). On the ‘nonsense’ substrate and the segments of the AAVS1-derived substrate away from target sites, subtle enrichments of dCas9 closely mirrored the distribution of PAM sites (two-sided Komolgorov-Smirnov test, significance level 5%) and dCas9 distribution on the ‘nonsense’ substrate more closely reflected the experimental PAM distribution than it did to 71.20% of 100 000 randomly generated sequences with the same dA, dT, dC and dG distributions (Supplementary Figure S2). As dCas9 binding along the ‘nonsense’ substrate (with 161 PAM sites in 1079 bp) corresponded so well with PAM site distribution, we interpreted this as a measurement of real dCas9-PAM interactions and estimated the mean single-site dissociation constant for dCas9 binding along the ‘non-specific’ substrate to be approximately 867 nM (standard deviation ± 209 nM). This can be understood as an estimate of the dCas9 binding dissociation constant on DNA with no protospacer homology.

### sgRNAs with a two nucleotide truncation at their 5′- ends (tru-gRNAs) do not increase binding specificity of dCas9 *in vitro*

Cas9 was found to still exhibit cleavage activity even if up to four nucleotides of the guide (protospacer-targeting) segment of the sgRNA (21) or crRNA (38) were truncated from their 5′-ends and Fu *et al*. (21) recently showed that use of sgRNAs with these 5′- truncations (optimally by 2–3 nucleotides) can actually result in orders-of-magnitude increase in Cas9 cleavage fidelity *in vivo*. It was suggested (21) that the increased sensitivity to mismatched sites (MM) using these truncated sgRNAs (termed ‘tru-gRNAs’, Figure 2A) was a result of its reduced binding energy between the guide RNA and protospacer sites. This implies that the binding energy imparted by the additional 5′- nucleotides on the sgRNA could compensate for any mismatched nucleotides and stabilize the Cas9 at incorrect sites, while the tru-gRNAs would be relatively less stable on the DNA if there are mismatches.

As a test of this proposed mechanism, we imaged dCas9 with a tru-gRNA with a two nucleotide 5′-truncation relative to the sgRNA used previously. The dCas9-tru-gRNA complexes were incubated with the engineered substrates that contained a series of full and partial protospacer sites. Again we find a distinct peak precisely at the full protospacer site (Figure 2C and Table 1), although the apparent association constant relative to dCas9 with a full sgRNA at this site decreases considerably (*i.e*. dissociation constant increases, see Table 2). However, relative to binding at full protospacer sites, off-target binding by dCas9 with the tru-gRNA at the protospacer sites with PAM-distal mismatches actually increases when compared to dCas9 with sgRNAs under these conditions (Figure 2C and Table 1). Similar to dCas9 with sgRNA, dCas9 with tru-gRNA binds to protospacers with either 10 or 5 PAM-distal mismatched sites with approximately equal propensities (note that as a result of the truncation the tru-gRNA is only expected to interact with the first 8 and 3 mismatches at those sites, respectively). These results suggest that increased cleavage fidelity using tru-gRNAs is not necessarily imparted by a relative reduction of binding propensity at off-target sites or a reduction in relative stability in the presence of mismatches. Rather, while there may be some ‘threshold’ effects where reduction of the association constant below ∼4–5 × 10^6^ M^-1^ effectively abolishes cleavage activity *in vivo*, these and additional results presented below suggest that the increased specificity exhibited by the tru-gRNAs may be influenced by discrimination in the cleavage mechanism itself. Furthermore, these findings would suggest that while tru-gRNAs can improve specificity in cleavage of active Cas9, they may not improve specificity in their binding activity for applications involving dCas9 (or chimeric derivatives) *in vivo*.

### Guide RNAs with 5′- hairpins complementary to their ‘PAM-distal’-targeting segments (hp-gRNAs) modulate the absolute binding propensity and profile of dCas9s bound to DNA with mismatched protospacers *in vitro*

We hypothesized that dCas9 specificity could be increased by extending the 5′- end of the sgRNA such that it formed a hairpin structure which overlapped the ‘PAM-distal’-targeting (or ‘non-seed’) segment of the sgRNA (Figure 2B). Similar topologies have been used recently for ‘dynamic DNA circuits’ which are driven by strand invasion (39,40). In those systems, the hairpins serve as kinetic barriers to invasion, with oligonucleotide invasion rates slowed several orders of magnitude in cases of attempted invasion by targets with mismatches (41). We hypothesized that, similarly, the hairpins here would be displaced during invasion of the full target sites, but inhibit invasion if there were mismatches between the target and the non-seed targeting region of the guide RNA (Figure 2B). In those cases, it may be more energetically favourable for the hairpins to remain closed. Previous efforts to add 5′- extensions to sgRNAs in order to complement additional nucleotides beyond the protospacer did not exhibit increased Cas9 cleavage specificity *in vivo*. Rather, they were found to be digested back approximately to their standard length in living cells (22). Based on the size and structure of the hairpin we anticipate that the hairpin could be accommodated within the DNA-binding channel of Cas9/dCas9 molecule and protected from degradation (35).

We generated sgRNAs with 5′- hairpins (hp-gRNAs) which overlapped the nucleotides complementary to the last six (hp6-gRNA) or ten (hp10-gRNA) PAM-distal positions of the protospacer. By mapping the observed binding locations of dCas9-hp-gRNAs on the engineered DNA substrate (Figure 2D), we again observe sharp peaks precisely at the protospacer site (PAM and protospacer located at sites 144–167, with binding peak at site 154.0 (95% confidence: 153.3–154.8) for dCas9-hp6-gRNA and at 158.3 (95% confidence: 157.6–158.9) for dCas9-hp10-gRNA). dCas9 with hp-gRNAs show a similar drop in affinity for the target site as with tru-gRNAs, however, in contrast to dCas9 with tru-gRNAs, dCas9 with hp-RNAs do not present any sharp binding peaks at off-target sites which would otherwise indicate strong, specific binding. We do note, however, that particularly with hp6-gRNA there is an enrichment of binding around the sites of protospacers with 5 or 10 mismatched PAM-distal sites. Because they lack the sharp binding peaks observed with sgRNA and tru-gRNA, these enrichments are not likely indicative of specific binding, but rather may indicate that the dCas9 had dissociated from these sites upon adsorption to the surface. This would indicate very weak binding at those off-target sites in the case of hp6-gRNA.

In the case of hp10-gRNA, binding to these mismatched sites is approximately at the level of the non-specific binding elsewhere on the substrate, representing a 22% decrease in the maximum observed off-target binding affinity relative to the tru-gRNAs (from to 3.18 × 10^6^ M^-1^ to 2.48 × 10^6^ M^-1^, Figure 2D) and a 57% decrease in maximum observed association constant at sites of protospacers with 10 PAM-distal mismatches. This increase in specificity of hp10-gRNA is also reflected by a similar binding dissociation constant as hp6-gRNA to the protospacer sites but a significant increase in the overall dissociation constant relative to the entire (specific + non-specific) engineered substrate (Table 2).

The distinct enrichment precisely at the complete protospacer sites suggests that upon invasion of full protospacer sites the hairpins in the hp-gRNAs are in fact opening, as the nucleotides which bind the PAM-distal sites of the protospacer would otherwise be trapped within the hairpin (39,40). A likely mechanism for the improvement of binding specificity is that, when unopened at protospacer sites with PAM-distal mismatches, the presence of the hairpin promotes melting of the guide RNA from these off-target sites (42). The results suggest that the hp-gRNAs can be used to tune Cas9/dCas9 binding affinities and specificity, and further manipulation of hairpin length, loop length and loop composition may allow for more fine control of these properties.

### Cas9 and dCas9 undergo a progressive structural transition as they bind to DNA sites that increasingly match the targeted protospacer sequence

It was observed using negative-stain transmission electron microscopy (TEM) (35) that, upon binding sgRNA, the structure of dCas9 compacts and rotates to open a putative DNA-binding channel between its two lobes. After binding to DNA containing the PAM and protospacer sequence, dCas9 undergoes a second structural reorientation to an expanded conformation. The role of this second transition was suggested to be related to strand invasion by sgRNA (43) or to align the two major Cas9 nuclease sites with the two separated DNA strands (35,44). However, these studies were performed only in the presence or absence of DNA containing fully-matched protospacer sequences, and examining the transition between these conformations at partially matched protospacer sites can provide insights into the mechanism of off-target binding and cleavage.

**Figure 3.**
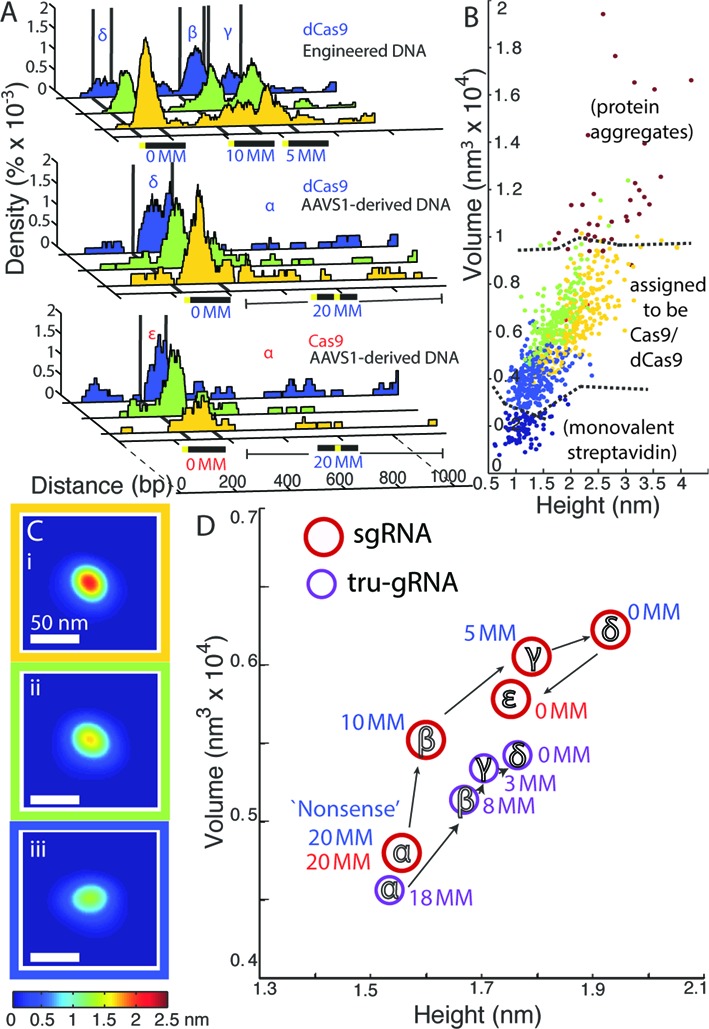
Cas9 undergoes a progressive conformational transition as it binds to sites that increasingly match the protospacer sequence. (**A**) Fraction of bound DNA occupied by Cas9/dCas9 along the DNA substrates, with colours representing populations of Cas9/dCas9 clustered according to their structures (by mean-squared difference after alignment, see text). Different features on DNA that were used for site-specific analysis of Cas9/Cas9 structural properties labelled as: non-specific sequences (α; ‘20 MM’), sites containing 10 PAM-distal mismatches within the protospacer (β, ‘10 MM’), sites containing 5 PAM-distal mismatches within the protospacer (γ, ‘5 MM’), or the full protospacer site (δ or ϵ for dCas9 or Cas9, respectively; ‘0 MM’). The ensemble average of the primary clusters are displayed below in (**C**) and colour-coded according to the clustered structures they represent. (**B**) Volume versus height of Cas9/dCas9 observed, colour-coded by the cluster to which each protein was assigned. Dashed lines delineate regions likely composed of aggregates (top right) or monovalent streptavidin adsorbed near DNA (bottom left). For comparison—mean height of streptavidin end-labels: 0.92 nm ± 0.006 nm (SEM); mean volume of streptavidin end-labels: 0.110 × 10^4^ nm^3^ ± 0.002 × 10^4^ nm^3^ (SEM); *n* = 1941. (**D**) Mean volumes and heights of Cas9/dCas9 with sgRNAs (red circles, with red labels for Cas9 and blue labels for dCas9) or tru-gRNAs (purple circles) bound at each feature on the substrates. Note that dCas9 with tru-gRNAs are only expected to interact the first 3 or 8 PAM-distal mismatches of the 5 MM and 10 MM sites (labelled ‘3 MM’ and ‘8 MM’ here, respectively). For standard errors of mean volumes and heights, see Table 2. For Cas9/dCas9 with sgRNAs, their structural properties at each feature are statistically distinct (δ – ϵ, α – ϵ: *P* < 0.05; α – β: *P* < 0.005; β – γ, γ – δ: *P* << 0.0005. Hotelling's T^2^ test).

Therefore, in addition to determining relative binding propensities, we also used AFM imaging to capture these putative conformational transitions by Cas9 and dCas9 as they bind to DNA at sites of various complementarity to the protospacer. We extracted the volumes and maximum topographical heights of Cas9 and dCas9 proteins with sgRNAs which appeared isolated on the DNA (*n* = 839) and mapped these values to their respective binding sites on DNA (Figure 3, Supplementary Figures S4 and S5). The binding site distribution is nearly identical to the distribution of the full data set, indicating that this selection was unbiased and representative. We then extracted the recorded image of each of these proteins (Supplementary Figure S4C–4D), aligned them pair-wise by iterative rotation, reflection, translation (28), and clustered (27) the protein structures according to their pair-wise mean-squared topographical difference (Supplementary Figure S5 and Table 3). See Supplementary Methods for more detail. A pronounced advantage of this technique is that it naturally clusters any monovalent streptavidin or any aggregated Cas9/dCas9 proteins that co-localize on the surface with the DNA separately from those assigned to be individual Cas9/dCas9 molecules, allowing for an unbiased analysis of the structural properties of these proteins on DNA. Analysis of the distribution of binding sites by either the putative streptavidin molecules or aggregated proteins reveals that they are both rare and uniformly distributed along the DNA and hence did not interfere with analysis of the binding site distributions (Supplementary Figure S5).

**Table 3.** Properties of dCas9/Cas9 with different guide RNA variants at fully-, partially- and non-complementary protospacer sites

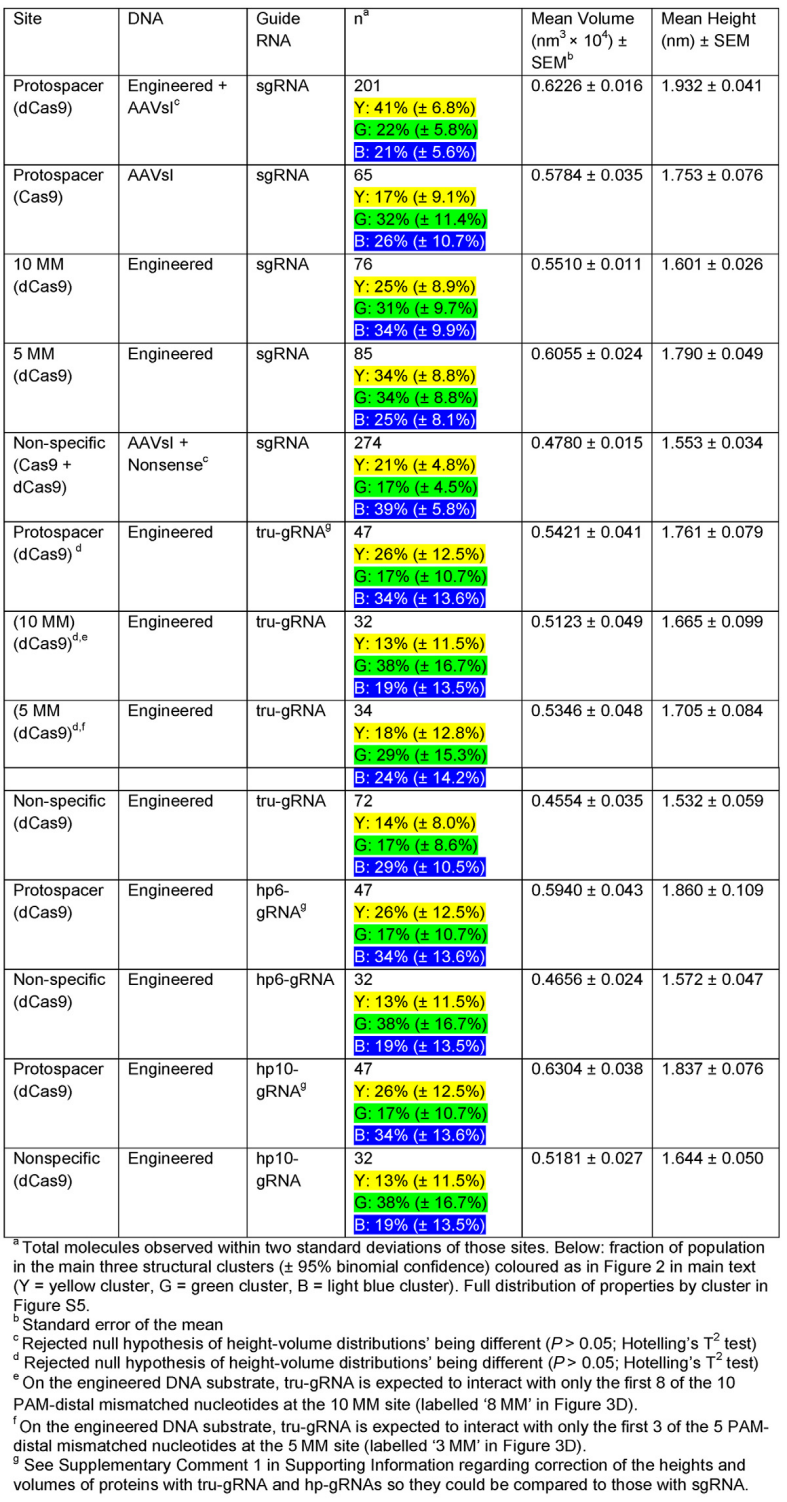					

At sites containing no homology to targets, such as on the ‘nonsense’ DNA substrate, dCas9 molecules with sgRNAs were predominately smaller and egg-shaped (Figure 3Ciii and Table 3). But as dCas9 proteins bind to increasingly complementary target sequences (Figure 3 (α − δ)), their height and volume significantly increase (Figure 3D and Supplementary Figure S5, Table 2) relative to non-specific binding, reaching a maximum size at the protospacer sequence. This increase in size is likewise accompanied by a shift in the population of dCas9 (Figure 3A, and Supplementary Figure S5, Table 2) from structures clustering with the flatter and egg-shaped conformations (Figure 3Cii and Ciii, blue and green) to those which increasingly cluster with slightly rounder structures possessing a large, central bulge (Figure 3Ci, yellow). This latter observed conformation is likely the expanded conformation previously observed *via* TEM and recently by size exclusion chromatography (45), and is presumably the active state where the nuclease domains of Cas9 are positioned properly around the DNA such that cleavage could occur most efficiently (45).

Catalytically active Cas9 undergoes a significant increase in size as it binds to the protospacer sequence as well (Figure 3(ϵ)); however there is a small, but statistically significant, decrease in size relative to dCas9, and the conformation of Cas9 at full protospacer sites tends to cluster with the flatter (green) structures. As we do not concurrently monitor whether the DNA has been cleaved at the time of imaging, it is unclear if this represents another conformational change after DNA cleavage or is a result of the mutational differences between Cas9 and dCas9; however as binding and strand invasion have been previously determined to be the rate-limiting steps (34,44) it is likely that the DNA within the Cas9 is cleaved during these measurements.

### Interactions between the guide RNA and the target DNA at or near the 16th protospacer site stabilize the Cas9/dCas9 conformational change

**Figure 4.**
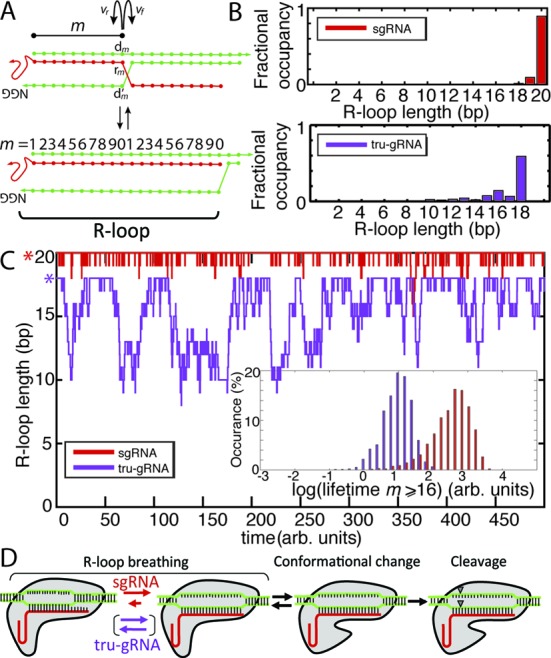
Kinetic Monte Carlo (KMC) experiments reveal differences in the stability of the R-loop, or the structure formed by the protospacer duplex with an invading guide RNA, within stably bound Cas9 for different guide RNA variants. (**A**) Schematic of strand invasion of the protospacer (green) by the guide RNA (red) for KMC experiments. The R-loop is highlighted. Transition rates for invasion (*v_f_* for the rate of *m* → *m* + 1, where *m* is the extent of the strand invasion or, equivalently, the length of the R-loop) or duplex re-annealing (*v_r_* for the rate of *m* → *m* − 1) are a function of the nearest-neighbour DNA:DNA and RNA:DNA hybridization energies. See text and Supplementary Methods for details. (**B**) Fractional time that the R-loop is of size *m* for sg-RNAs (red) or tru-gRNA (purple) derived from KMC experiments ‘at equilibrium’ (simulation initiated at *m* = 20 or 18, respectively). Simulation run until *t* ≥ 10 000 (arbitrary units). (**C**) Kinetic Monte Carlo time course of the R-loop ‘breathing’ for sgRNA (red) and tru-gRNA (purple) after full invasion (simulation initiated at *m* = 20 or 18, respectively). Asterisks highlight the starting position for the simulation. (inset) Histogram of the respective lifetimes to transit from full invasion to *m* < the 16th position. (**D**) Proposed model for the mechanisms governing Cas9/dCas9 specificity, based on results of AFM imaging and KMC experiments (see main text). Cas9/dCas9 binds to the PAM and the guide RNA invades into the PAM-adjacent protospacer duplex. During this strand invasion, the guide RNA must displace the complementary strand of the protospacer. Competition between invasion and re-annealing of the duplex results in a dynamic (‘breathing’) R-loop structure. The stability of the 14th–17th sites of the protospacer-guide RNA interaction, which is dramatically increased by binding at the 19th and 20th sites, promotes a conformational change in the Cas9/dCas9 that authorizes DNA cleavage in Cas9.

AFM imaging directly reveals that although dCas9/Cas9 retains a significant propensity to bind protospacer sites with up to 10 distal mismatches, binding to DNA sites that are increasingly complementary to the protospacer drives an increasing shift in the population of dCas9/Cas9 proteins towards what appear to be the active conformation. Notably, we see similar shifts in structures between off-target sites and perfectly-matched sites for dCas9 with hp-gRNAs as well (Table 3 and Supplementary Figure S6). The presence of complementary PAM-distal sequences is known to be associated with increased stability of Cas9 on DNA (44). It was also recently found that Cas9 binding to single-stranded DNA with increasing PAM-distal complementarity to the protospacer (from 10 to 20 sites) resulted in an increase of the size of the protein-RNA-DNA complex. This was also then associated with a transition of Cas9 activity from nicking behaviour to full cleavage (45). Here, we directly can determine the volumes of Cas9/dCas9 bound onto double-stranded DNA sites. An analysis of the structural properties of individual Cas9/dCas9 proteins on double-stranded DNA reveals a steady conformational transition with increasingly matched target sequences that is consistent with a ‘conformational gating’ mechanism, where sgRNA base-pairing with these distal sites also stabilizes the active conformation so that efficient cleavage may occur, whereas binding to sites with numerous distal mismatches shifts the equilibrium away from the active structure (*i.e*. see Figure 4D).

Along these lines, we see this effect is dramatically muted for dCas9 with the tru-gRNA (Figure 3D and Table 3), with a smaller shifts between the structural populations within which the proteins cluster (Supplementary Figure S6). Additionally, while we see a statistical difference between the height-volume properties of dCas9-tru-gRNAs that are non-specifically bound and those bound at full or partial protospacer sites (*P* < 0.05; Hotelling's T^2^ test), at sites that increasingly match the protospacer (10 MM, 5 MM, and full protospacer sites) their structural properties are not statistically differentiable (Figure 3D and Table 3). It was recently postulated that while invasion of the first 10 bp of the protospacer initiates a conformational change in Cas9, full invasion of the protospacer by the guide RNA helps to drive a further shift to the complete active state (45). We therefore hypothesized the observed depression of the conformational change at increasingly matched protospacer sites for dCas9 with tru-gRNAs (relative to those with sgRNAs) was a result of the decreased stability of these guide RNAs at PAM-distal sites.

To investigate the relative stabilities of sgRNAs and tru-gRNAs at these sites, we performed a KMC study of the dynamic structure of the R-loop—*i.e*. the structure formed by the invading guide RNA bound to a segment of contiguous DNA, exposing a single-stranded loop of the that segment's complementary DNA (44) (Figure 4A and Supplementary Figure S7)—during and after strand invasion. See Supplementary Methods for more detail. Briefly, using a Gillespie-type algorithm (29), we modelled the strand invasion of the guide RNA bound up to protospacer site *m* as a sequential, nucleotide-by-nucleotide competition between invasion (breaking of base-pairing between the protospacer and its complementary DNA strand, then replacement with a protospacer-guide RNA base-pair) and re-annealing (the reverse), with sequence-dependent rates *v_f_* and *v_r_* for invasion and re-annealing, respectively (Figure 4A). To first-order, we approximate the transition rate from state *m* to *m* + 1, *v_f_*, to be proportional to exp (−(ΔG°(*m* +1)_RNA:DNA_ – ΔG°(*m* +1)_DNA:DNA_) / 2RT), where ΔG°(*m* + 1)_RNA:DNA_ is free energy of the base-pairing between the RNA and protospacer at site *m* + 1 and ΔG°(*m* + 1)_DNA:DNA_ is the free energy of the base-pairing between the protospacer and its complementary DNA strand at *m* + 1 (R is the ideal gas constant, T is the temperature, and the 1/2 term is added to satisfy detailed balance). *v_r_* is estimated similarly as proportional to exp (−(ΔG°(*m*)_DNA:DNA_ – ΔG°(*m*)_RNA:DNA_)/2RT). Transition rates of this type have been previously used for computational studies of nucleotide base-pairing and stability (46,47), and here they allowed us to capture the general dynamics of the R-loop in a sequence-dependent manner.

In general, RNA:DNA base-pairs are energetically stronger than DNA:DNA base-pairs (48), and at equilibrium we see from the KMC trajectories that the guide RNAs are stably bound to the protospacer, as expected (Figure 4C). However, while sgRNA is quite stable and remains nearly totally invaded—during 95% of simulated time course, the strand remains invaded up to the 19th protospacer site (Figure 4B)—tru-gRNA exhibits significant fluctuations of protospacer re-annealing at PAM-distal sites (Figure 4B and C). Because the only difference between the dCas9-sgRNA and dCas9-tru-gRNA is a simple truncation of two 5′- nucleotides from the guide RNA, and because we see an inhibition of the conformational change by dCas9-sgRNA at sites containing 5 PAM-distal mismatches, these results suggest that the conformational change to a fully active state is stabilized by interactions between the guide RNA and protospacer near the 16th site of the protospacer, which is disrupted by the instability of the tru-gRNA in that region. In fact, the KMC experiments show that the mean lifetime between full invasion and re-annealing of the DNA back to the 16th site is decreased by two orders of magnitude when replacing the sgRNA with the tru-gRNA (Figure 4C inset). This result is consistent with the earlier finding that while Cas9 activity with tru-gRNA variants with two or three nucleotide (nt) truncations was modulated depending on sequence context, and that cleavage in all tested cases was dramatically reduced by ∼90–100% by 4 nt truncations and abolished after a 5 nt truncation (21,38). Our conclusion—that the conformational change to the protein activate state is stabilized by these interactions at or near the 16th site of the protospacer—is further supported by our finding that sgRNA stability at the 14th–17th protospacer positions, which we estimate from additional KMC experiments described below, is correlated with experimental off-target cleavage *in vivo* (see below) while stability of the sgRNA at protospacer positions 18–20 is not.

### Fluctuations of the guide RNA-protospacer R-loop suggest a mechanism of mismatch tolerance by Cas9/dCas9 and of increased specificity in cleavage by tru-gRNAs

**Figure 5.**
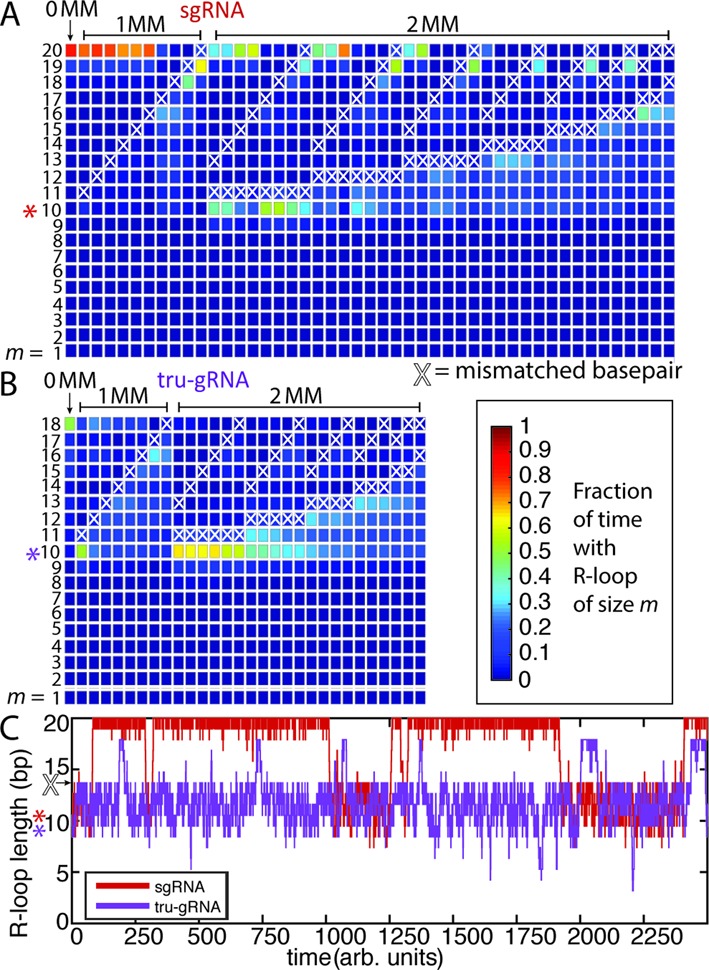
KMC experiments reveal differences in ability to traverse mismatches (MM) and invade the protospacer depending on guide RNA structure. (**A**–**B**) Fractional occupancy by time of R-loop lengths *m* for (**A**) sgRNA or (**B**) tru-gRNA during invasion derived from KMC experiments (initiated at *m =* 10, highlighted by asterisk). White X's indicate positions of mismatches. Simulation run until *t* ≥ 10 000 (arbitrary units) and the results are averaged over 100 trials. (**C**) Representative KMC time courses for strand invasion (starting at *m* = 10) with a mismatched site at *m* = 14 (arrow) for sgRNA (red) and tru-gRNA (purple). While sgRNAs are largely stably invaded after bypassing a mismatch, tru-gRNAs are repeatedly re-trapped behind the mismatch as a result of the inherent volatility of their R-loops (see Figure 4).

To investigate mechanisms by which Cas9 or dCas9 can tolerate or become sensitized to internal mismatches in protospacers, we performed a series of KMC experiments again using the AAVS1 protospacer sites, but now where one or two PAM-distal (≥10 bp away from the PAM) mismatches were introduced (Figure 5). Cas9 is generally more tolerant of PAM-distal mismatches than PAM-proximal mismatches (13–15,18). However, Hsu *et al*. (18) identified significant and varying differences in estimated Cas9 cleavage rates at protospacers containing PAM-distal mismatches depending on sequence context, type of mismatch, and site of the mismatch. Based on our AFM and earlier KMC experiments, we hypothesized the differences in cleavage rates may similarly be a result of different stabilities of the guide RNA near the 16th site of the protospacer. For these simulations, we only examined sequences with protospacers-guide RNA pairs which would result in isolated rG·dG, rC·dC, rA·dA and rU·dT mismatches, for which the sequence context-dependent thermodynamic data are the most complete (32) and suitable for our KMC model. The effects of these mismatched base-pairs are not expected to lower the overall binding energy between the sgRNA and protospacer dramatically (Supplementary Table S1); *e.g*. single rG·dG, rC·dC, rA·dA and rU·dT mismatches lower RNA:DNA melting temperatures on average by 1.7°C (32). Rather, their effect is expected to be kinetic rather than thermodynamic in nature by hindering strand displacement at the mismatch (49,50). Hence we initiated the KMC experiments as proceeding from the 10th protospacer site (initial R-loop length *m* = 10), such as would be occurring during strand invasion.

KMC experiments were then performed to investigate the kinetics of strand invasion in the presence of PAM-distal mismatches. In all cases (1000 trials each), both the sgRNAs and the tru-gRNAs remain quite stably bound even when there are mismatches (*i.e*. are not observed to completely melt off) and are often able to quickly bypass these positions to complete full invasion (Figure 5C and Supplementary Figure S8), although the mean first passage time for total strand invasion varied significantly depending on the position of the mismatch site (Supplementary Figure S8). The R-loops formed by sgRNAs are quite stable during invasion (Figure 5A), as the sgRNAs are often able to remain fully invaded even in the presence of multiple mismatches. The results qualitatively resemble those of earlier *in vitro* studies of dCas9/Cas9 binding and cleavage on mismatched targets (38). However, in the case of tru-gRNAs (Figure 5B), the R-loops are often trapped behind the position of the mismatch. The mean first passage time across mismatches is similar for both sgRNAs and tru-gRNAs (Supplementary Figure S8), but an inspection of the time courses for the KMC reveals that, because of the inherent volatility of the R-loop for tru-gRNAs, tru-gRNAs are often quickly ‘re-trapped’ behind the mismatch (Figure 5C). For sgRNAs, this re-trapping is much less frequent. Hence, in combination with AFM imaging, the results of the KMC experiments suggest that the origin of increased tru-gRNA specificity lies not in discrimination during binding but rather in the volatility of its R-loop (Figure 4D) such that it becomes repeatedly trapped behind mismatches even after initially bypassing them, making Cas9 less likely to assume the active conformation. For sgRNAs, once a mismatch is bypassed it can remain fully invaded with relatively little perturbation, suggesting a mechanism of mismatch tolerance.

### Stabilities of the guide RNA interaction with the 14th–17th positions of the protospacer are correlated with experimental off-target Cas9 cleavage rates, while overall guide RNA–protospacer binding energies are not

To verify whether the stabilities of the R-loop at or near the 16th position of the protospacer—which was implicated by AFM studies to be connected to the conformational change in Cas9—are associated with Cas9 activity *in vivo*, we performed a KMC analysis of R-loop stability on the sequences used by Hsu *et al*. (18). The data set of Hsu *et al*. (18) consisted of measurements of the cleavage frequency at 15 different protospacer targets containing various point mutations versus the guide RNA that were performed to investigate cleavage specificity by Cas9. This data set contained 136 protospacer-guide RNA pairs that possessed a single, isolated mismatch of type rG·dG, rC·dC, rA·dA and rU·dT in the PAM-distal region (Supplementary Table S1), which we investigated using KMC simulations initiated at R-loop size *m* = 10 to model strand invasion. The inclusion of a single mismatched site from this set decreased the magnitude of their overall guide RNA–protospacer binding free energy on average by about only 6% relative to perfectly matched targets although, as mentioned, there was a wide distribution Cas9 cutting frequencies observed for these guide-RNA protospacer pairs whose origin was not obvious (18).

**Figure 6.**
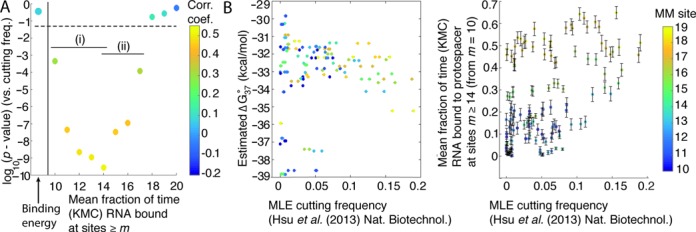
Experimental (Hsu *et al*. (2013) (18)) cutting frequencies at target sites containing a single rG·dG, rC·dC, rA·dA and rU·dT mismatch in the PAM-distal region (≥10th protospacer site) are correlated with stabilities of the R-loop determined from KMC experiments. (**A**) log_10_(*P*-value) of the correlations between Cas9 cutting frequency and stability of R-loop at sites *m* (fraction of time the guide RNA remains bound to the protospacer at site *m*, see text) during strand invasion initiated at site *m* = 10. (i) Stability at sites *m* = 10 to *m* = 14 is highly anti-correlated with the probability that the guide RNA will fall off the protospacer prior to traversing the mismatch (Supplementary Figure S9B), while (ii) sites m = 14 to m = 17 are associated from AFM images with the conformational change which induces cleavage activity. Colour corresponds to the correlation coefficient. (**B**) Experimental cutting frequency does not correlate significantly with estimated guide RNA–protospacer equilibrium binding free energies (ΔG°_37_) (left), while it does with stability of site *m* = 14 during strand invasion (right). Error bars are standard errors of the mean times site *m* = 14 is bound by the guide RNAs. For these kinetic Monte Carlo experiments, max(*t*) = 100 (arbitrary units). Colour bar is used to show the location of the mismatched (MM) site.

The mean fraction of time the RNA was bound stably to each site of the protospacer was determined for each guide RNA over 1000 trials, which was then correlated to the maximum-likelihood estimated cleavage activity of Cas9 presented in Ref (18) (Table 4, Figure 6 and Supplementary Figure S9). We found a moderate (0.433) but statistically significant (*P* < 1 × 10^−6^) correlation between guide RNA stability at the 16th protospacer position and reported off-target cleavage activity. Notably, no statistically significant correlation was found between cleavage rate and the predicted DNA:RNA binding energies alone (0.0786; *P* = 0.3631) (Figure 6A and B). In addition to R-loop stability at the 16th position, a significant correlation is also found for stability the 17th protospacer site and reported cleavage (Table 4), but this was not the case for sites ≥18th site (Figure 6). While the KMC model presented here is based on a relatively simple model of strand invasion, these results further suggest that stability of the 16th–17th positions of the protospacer, and hence the concomitant conformational changes we observed, are associated with Cas9 cleavage activity *in vivo* (Figure 4D).

**Table 4.** Correlations between experimental (Hsu *et al*. (2013) (18)) cutting frequencies at target sites containing a single rG·dG, rC·dC, rA·dA and rU·dT mismatch in the PAM-distal region (≥10th protospacer site)^a^ and measures of guide RNA–protospacer stability

		log_10_(*P*-value)	Correlation coefficient
Hsu *et al*. (18) estimated cutting frequency versus guide RNA–protospacer binding energy^b^		−0.4400	(0.0786)
Hsu *et al*. (18) estimated cutting frequency versus position of mismatch		−5.8258	0.3990
Hsu *et al*. (18) estimated cutting frequency versus fractional time guide RNA bound at position ≥the *m*th protospacer site in a simulated R-loop (KMC)^c^	*m* = 14	−9.5550	0.5078
	*m* = 15	−7.4854	0.4522
	*m* = 16	−6.9510	0.4333
	*m* = 17	−3.9270	0.3191
	*m* = 18	−0.7639	(0.1159)
	*m* = 19	−0.5546	(0.1058)
	*m* = 20	−0.2346	(−0.0176)

^a^*n* = 136.

^b^See Supplementary Methods and Supplementary Table S1 for details.

^c^See text for details. Max(*t*) = 100.

We limited most of our analysis to interactions with the 16th–18th nucleotides of the protospacer because of the observed structural differences between dCas9 with tru-gRNAs and sgRNA by AFM. However, we also observe an increase of the strength and statistical significance of the correlations between cleavage and the stability of the 14th and 15th protospacer sites (Figure 6A), with greatest significance for the correlation at the 14th site. Because the R-loop is a dynamic structure (44,50) (Figure 4D), it is possible that interactions with these sites are those critical ones believed to be responsible for DNA cleavage. That is, truncation of the guide RNA by four or five nucleotides may abolish cleavage activity by sufficiently destabilizing the R-loop at the 14th or 15th position in much the same way that the tru-gRNA destabilized the R-loop at the 16th–17th positions of the protospacer. However, because in our model 14th and 15th positions are necessarily invaded whenever the 16th position is bound by sgRNA, it is likely that these positions are additionally informative because they are also more strongly anti-correlated with the probability of sgRNA dissociation from the duplex prior to bypassing the mismatched site (Figure 6Ai and Supplementary Figure S9B), another mechanism by which cleavage would fail to occur. At present, there is no crystallographic evidence which directly relates strand invasion to the observed conformational change believed to authorize cleavage. However, based on the evidence provided by AFM experiments presented here and the results of the kinetic Monte Carlo simulations, we conclude that stability of the guide gRNA at the 14th–17th positions of the protospacer during invasion is critical for this conformational change and, ultimately, the specificity of Cas9 cleavage.

(Furthermore, we will also note that the binding distribution of dCas9-sgRNAs that we observed *via* AFM on the engineered DNA substrates (*i.e*. Figure 1D) can be approximately described using this model of strand invasion (Supplementary Figure S7): the estimated dissociation rates of dCas9 at protospacer sites with 10 or 5 contiguous PAM-distal mismatches (10 MM and 5 MM sites) are calculated to be significantly lower than those at sites with 15 PAM-distal mismatches (15 MM) but within an order of magnitude of one another other. These results would suggest that overall off-target binding propensity is largely determined by the lifetime of protospacer-guide RNA interaction, while cleavage depends on the internal dynamics of the R-loop and stability of the guide RNA at PAM-distal sites.)

These results underscore the fact that an understanding of the R-loop as a dynamic structure (44,50) in competition between strand invasion and DNA re-annealing can be useful in understanding mechanisms of off-target cleavage and mismatch tolerance. As mentioned, no statistically significant correlation was found between cleavage rate and the predicted DNA:RNA binding energies alone (Figure 6B), suggesting that the kinetics of strand invasion should be considered when attempting to determine Cas9 activity at off-target sites. An interesting recent finding is that while cleavage is abolished when four or five nucleotides are truncated from the guide RNA (21,38), Cas9 is still able to cleave DNA with up to six contiguous distal-mismatch sites (1,45). This would suggest that transient, non-specific interactions at these PAM-distal sites could sufficiently stabilize the conformational shifts necessary for cleavage. Since we see minority populations of dCas9-sgRNA at partial protospacer sites with similar structures to those at the full protospacer (yellow, Figure 3Ci), this population may represent the fraction of Cas9 in a transiently-stabilized active conformation. As such, this population may be responsible for off-target cleavage.

## CONCLUSIONS

**Figure 7.**
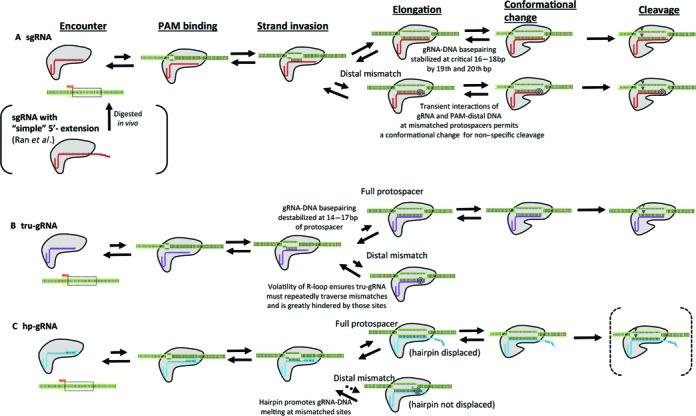
Summary of proposed mechanisms by which the structure of the guide RNA affects Cas9/dCas9 specificity. (**A**) For the single guide RNA (sgRNA), the first few nucleotides of the RNA (which bind to the 18th–20th positions of the protospacer) stabilize R-loop breathing and binding at the 14th–17th sites of the protospacer, allowing for efficient conformational transitions to the active state in order to permit cleavage. However, this increased stability imparted by these bases allows for transient stabilization at mismatched sites and the conformational change permitting cleavage. In many cases, having traversed a mismatch, R-loops remain stably fully-invaded. (**B**) For guide RNAs with the first few (here two) nucleotides truncated (tru-gRNA), the reduced stability of the R-loop (characterized by significant volatility) decreases the probability of maintaining the active conformation. When there are mismatched sites in the protospacer, the volatility of the R-loop ensures that it will becomes quickly and repeatedly ‘re-trapped’ behind the mismatch and greatly hindered at those sites. (**C**) While ‘simple’ extensions of the 5′- end of the guide RNA to target the protospacer and adjoining sites beyond the protospacer was found to be digested back to approximately sgRNA length *in vivo* (13) (Figure 7A), guide RNAs with 5′- hairpins complementary to ‘PAM-distal’-targeting segments (hp-gRNAs) are anticipated to remain protected within the structure of the Cas9/dCas9 prior to invasion. After binding a PAM site and initiating strand invasion by the hp-gRNA, upon binding to a full protospacer the hairpin is opened and full strand invasion can occur. If there are PAM-distal mismatches at the target site, then it is more energetically favourable for the hairpin to remain closed and strand invasion is hindered. Testing and optimization of the activity of nuclease-active Cas9s with hp-gRNAs *in vivo* is presently underway.

The results presented here show that while Cas9/dCas9 binding specificity is largely determined by interactions with the PAM-proximal region, DNA cleavage specificity is likely governed by a conformational change to an activated structure that is stabilized by guide RNA interactions at the 14th–17th bp region of the protospacer (Figure 4D). KMC experiments reveal that the R-loop formed during strand invasion of the guide RNA can be quite a dynamic structure even when the guide RNA remains stably bound, which suggests a mechanism for the improved specificity of tru-gRNAs, and an origin of off-target cleavage *via* transient stability of the guide RNA-protospacer at the critical region around mismatched sites. The proposed mechanisms for the effects of each of the sgRNA variants on Cas9/dCas9 specificity are summarized in Figure 7; in the future, single-molecule fluorescence resonant energy transfer (FRET) studies of strand invasion by Cas9 can help to confirm and clarify these mechanisms.

Using AFM, we found that hp-gRNAs significantly weakened or abolished specific binding at homeologous targets. Though we are currently testing their stability *in vivo* and their activity with nuclease-active Cas9, hp-gRNAs may be valuable for modulating dCas9 binding affinity and specificity in their potential applications in biology and medicine. Specifically, based on the narrow geometry of the Cas9 binding channel (35), the presence of an unopened hairpin at mismatched protospacers may inhibit the conformational change by Cas9 to the active state. The opening of the hairpin in hp-gRNAs upon binding could also be used as a binding-dependent signal *in vivo* (4), *e.g*. to nucleate dynamic DNA/RNA structures only upon binding to specific sites (39).

Earlier guide RNA truncation studies raised the question of why do natural Cas9 systems employ a crRNA which targets 20 bp protospacer sites when only a guide sequence of 16 nucleotides is required for cleavage and the additional nucleotides (>18) do not improve cleavage specificity *in vivo*. These results suggest that presence of the ‘extra’ 5′- nucleotides which bind to the 19th and 20th protospacer sites buffer this transient re-annealing at the critical 14th–17th sites of the protospacer, allowing efficient conformational change to the active state and subsequent cleavage to occur. Unlike the Cascade complex of Type IE CRISPR-Cas systems, Cas9 does not ‘lock’ and stabilize the R-loop into a long-lived structure upon full invasion (45). The results of AFM and KMC experiments suggest that stability of the guide RNA at these sites shifts the equilibrium structure of Cas9 towards the active conformation upon full invasion (Figure 4A), while the volatility of R-loops for ‘truncated’ guide RNAs reduces the pressure to shift the equilibrium to the active state. The promiscuous activity of Cas9 with sgRNAs versus tru-gRNAs might also hold evolutionary advantages in its role as an agent of adaptive immunity in prokaryotes to invasive DNA (1,51), since the DNA of invading phages undergo rapid point mutations at sites targeted by Cas9 in order to avoid cleavage (52,53).

The design of guide RNA sequences for Cas9/dCas9 applications *in vivo* has focused primarily on avoiding targets with multiple sites having similar sequences in the genome. However, a recent study exploring off-target cleavage found that current methods for predicting off-target activity were largely ineffective (17). Since we found the stability of the R-loop during invasion correlates with off-target cleavage rates significantly better (Table 4) than guide RNA-protospacer binding energies alone or the position of the mismatch—another important criteria used in guide RNA design—an increased understanding of R-loop dynamics and DNA:RNA interactions can inform the better design specific guide RNAs sequences. Additionally, the stability of the R-loop at shorter times after the initiation of invasion was correlated with experimental cleavage rate much better than was the long-term stability in the KMC experiments (Supplementary Figure S9A), suggesting that the kinetics of strand invasion will also prove to be a critical factor in off-target activity prediction. However, the findings presented here are limited with respect to the multiple RNA:DNA mis-pairs tolerated by Cas9 (15,17,18), for which we were constrained by the availability of thermodynamic data. This limitation is a result of the energetics of RNA:DNA base-pairing being complicated by a number of relatively stable ‘wobble’ base-pairings with sequence-dependent binding energies (32,54), not all of which have been rigorously characterized and some of which behave counterintuitively. For example, in some sequence contexts, certain mis-pairs such as rG·dG actually stabilize the RNA:DNA duplex (32,54). Additionally, the energetic effects of multiple, consecutive mis-pairs between RNA and DNA, which are often identified in assays of off-target activity by Cas9, have not been well characterized. More sophisticated models that incorporate improved energetic details, guide RNA:DNA secondary structure, and/or the effects of CpG meythlation (which is also known to affect Cas9 binding (16)) can likely improve the prediction of off-target cleavage in this way. Alternatively, applying protein engineering (55) to disrupt the stability of Cas9 to assume the active conformation until a complete protospacer is bound may have a similar effect as the tru-gRNA in improving specificity *in vivo*.

## Supplementary Material

SUPPLEMENTARY DATA
