# Structure and specificity of the RNA-guided endonuclease Cas9 during DNA interrogation, target binding and cleavage

**DOI:** 10.1093/nar/gkv1293

**Published:** 2015-11-17

**Authors:** Eric A. Josephs, D. Dewran Kocak, Christopher J. Fitzgibbon, Joshua McMenemy, Charles A. Gersbach, Piotr E. Marszalek

**Affiliations:** 1 Department of Mechanical Engineering and Materials Science, Edmund T. Pratt, Jr. School of Engineering, Duke University, Durham, NC 27708, USA; 2 Department of Biomedical Engineering, Edmund T. Pratt, Jr. School of Engineering, Duke University, Durham, NC 27708, USA; 3 Center for Genomic and Computational Biology, Duke University, Durham, NC 27708, USA; 4 Department of Orthopaedic Surgery, Duke University Medical Center, Durham, NC 27710, USA

*Nucl. Acids Res*. 43 (18): 8924–8941. doi: 10.1093/nar/gkv892

The authors wish to make the following corrections to their article:

Under the section ‘Atomic Force Microscopy’ of Materials and Methods, the authors accidentally stated the use of the chemical 3-aminopropylsiloxane. The correct chemical should have been 1-(3-aminopropyl)silatrane.

The results and conclusion of the article are not affected and remain valid. The authors apologise to the readers for the inconvenience caused.

