# Supplementary material for: Structure and specificity of the RNA-guided endonuclease Cas9 during DNA interrogation, target binding and cleavage
Source: Nucleic Acids Res. 2015 Oct 10;43(18):8924–41. doi: 10.1093/nar/gkv892 (PMC4605321; doi:10.1093/nar/gkv892)
Supplement: SUPPLEMENTARY DATA [file supp_43_18_8924__index.html]

Structure and specificity of the RNA-guided endonuclease Cas9 during DNA interrogation, target binding and cleavage — Structure and specificity of the RNA-guided endonuclease Cas9 during DNA interrogation, target binding and cleavage — SUPPLEMENTARY DATA 

# Structure and specificity of the RNA-guided endonuclease Cas9 during DNA interrogation, target binding and cleavage

## SUPPLEMENTARY DATA

- SUPPLEMENTARY DATA
