# Supplementary material for: Structure and specificity of the RNA-guided endonuclease Cas9 during DNA interrogation, target binding and cleavage
Source: Nucleic Acids Res. 2015 Oct 10;43(18):8924–41. doi: 10.1093/nar/gkv892 (PMC4605321; doi:10.1093/nar/gkv892)
Supplement: SUPPLEMENTARY DATA [file supp_gkv892_nar-01958-h-2015-File009.docx]

**Structure and Specificity of the RNA-guided Endonuclease Cas9 during DNA Interrogation, Target Binding, and Cleavage**

Eric A. Josephs,* D. Dewran Kocak, Christopher J. Fitzgibbon, Joshua McMenemy, Charles A. Gersbach, and Piotr E. Marszalek*

**Supporting Information**

Supplementary Methods

3

Supplementary Comment 1: Correction of dCas9-tru-gRNA and dCas9-hp-gRNA data for comparison with dCas9-sgRNA structural properties

7

Figure S1: Purity of expressed Cas9 and dCas9

8

Figure S2: Additional Images of Cas9 / dCas9 bound to DNA

9

Figure S3: Binding to ‘nonsense’ substrate containing no homology (>3 bp) to protospacer sequence

10

Figure S4: Representative figure of dCas9-sgRNA bound to RNA and example of processing of protein structural properties

11

Figure S5: Properties of Cas9/dCas9-sgRNAs mapped to their respective binding sites

12

Figure S6: Structural properties of Cas9/dCas9 with tru-gRNA and hp-gRNAs at their respective binding sites

13

Figure S7: Model of the strand invasion of DNA protospacers by guide RNAs, and estimated binding stabilities of RNA invaded into protospacers with PAM-distal mismatches

14

Figure S8: Simulated mean first passage times to traverse the mismatched site during strand invasion by sgRNA and tru-gRNA

15

Figure S9: Correlations between Cas9 cleavage frequency (Hsu *et al. (*2013)) and measures of R-loop stability derived from kinetic Monte Carlo.

16

Table S1: DNA, guide RNA sequences, and estimated cutting frequencies from Hsu *et al.* (2013) used for correlation analysis

17

DNA/RNA Sequences

21

References

23

***Supplementary Methods****:*

*Cloning of Cas9, dCas9, and sgRNA Expression Plasmids*

The plasmids encoding wild-type Cas9 and dCas9 were obtained from Addgene (plasmid 39312 and plasmid 47106). Plasmids for the expression of Cas9 and dCas9 in bacteria were cloned using Gateway Cloning (Life Technologies). Briefly, PCR was used to amplify Cas9 and dCas9 genes and to add flanking attL1 and attL2 sites. BP recombination was performed to transfer these genes to a shuttle vector, after which LP recombination was performed to transfer these genes to pDest17, which adds an N-terminal hexa-histidine tag (Life Technologies). The plasmids encoding the chimeric sgRNA and sgRNA variants (described below) were cloned as previously described (1).

*Expression and Purification of Cas9, dCas9*

Plasmids encoding Cas9 or dCas9 were transformed into SoluBL21 competent cells (Genlantis) according to standard techniques (2). Single colonies were used to inoculate 25 mL starter cultures. 25 mL starter cultures were grown overnight and used to inoculate 1 L cultures. Inoculated 1L cultures were grown for 5 hours at 25 °C after which the temperature was dropped to 16 °C and protein expression induced by the addition of 0.1 mM IPTG. Induced cultures were grown for another 12 hours at 16 °C. Cells were harvested by centrifugation at 4000x g and stored at -80 °C for long-term storage.

Cell pellets were resuspended in 30 mL of Lysis Buffer (50mM Tris-HCl, 500 mM NaCl, 10 mM MgCl_2_, 10 % v/v glycerol, 0.2% Triton-1000, and 1mM PMSF). The cell suspension was lysed by sonication at 30% duty cycle for 5 minutes. The suspension was then centrifuged for 30 minutes at 12,000xg. The supernatant was then taken and incubated with Ni-NTA resin (Qiagen) for 30 minutes under gentle agitation. The resin was then loaded onto a column, washed with Wash Buffer (35 mM imidizole, 50mM Tris-HCl, 500 mM NaCl, 10 mM MgCl_2_, 10 % v/v glycerol), and eluted with Elution Buffer (120 mM imidizole, 50mM Tris-HCl, 500 mM NaCl, 10 mM MgCl_2_, 10 % v/v glycerol). Ultracel-30k centrifugal filters were then used to exchange solvents to the Storage Buffer (50mM Tris-HCl, 500 mM NaCl, 10 mM MgCl_2_, 10% v/v glycerol). The samples were then aliquoted and frozen at -80 °C. Representative polyacrylamide SDS gels of purified Cas9 and dCas9 are presented in Figure S1, indicating approximately >95% purity.

*Expression and purification of sgRNA and guide RNA variants*

Guide RNAs were *in vitro* transcribed using the MEGAshortscript T7 Transcription Kit (Life Technologies. DNA templates with a T7 promoter were generated via PCR from guide RNA plasmids and reactions were set up following the manufacturer’s instructions. The T7 templates for the guide RNAs with 2 nucleotides truncated from their 5’- ends (tru-gRNAs) and those with 5’ extensions that form hairpins (hp-gRNAs) were generated by PCR off of the standard gRNA plasmids. The RNA was then purified using phenol-chloroform extraction using standard techniques (2).

*Preparation of DNA substrates*

Genomic DNA was extracted and purified from HEK293T cell line using the DNeasy kit (Qiagen), following the manufacturer's protocol. The AAVS1 locus was then amplified using PCR. The 1198 bp AAVS1-derived substrate was constructed via direct PCR from genomic DNA using primers from Integrated DNA Technologies (IDT): 5’-\Bt\-CCAGGATCAGTGAAACGCAC-3’ and 5’-GAGCTCTACTGGCTTCTGCG-3’, where \Bt\ represents a biotinylation of the primer at the 5’- end. The ‘engineered’ DNA substrate, which contains a series of PAMs and full or partial protospacer sites, was ordered as two gBlock fragments each containing an EcoRI restriction site on one end. Substrates were digested, ligated together, and then enriched *via* PCR with primers (Integrated DNA Technologies, IDT): 5’-\Bt\-CATGACGTGCAGCAAGC-3’ and 5’-CGACGATGCGCTGAATC-3’. To construct a ‘nonsense’ substrate containing no sites exhibiting homology (greater than 3 bp) to the protospacer: a 690 bp DNA construct was synthesized (GeneScript, Inc.) containing a series of restriction sites, and an addition length of DNA from lambda DNA (New England Biolabs) was sub-cloned into the construct; the 1078 bp substrate was then PCR amplified using primers (IDT): 5’-\Bt\-GACCTGCAGGCATGCAAGCTTGG-3’ and 5’- CAGCGTCCCCGGTTGTGAATCT-3’. All DNA was gel purified, diluted to 25 nM in working buffer (20 mM Tris-HCl (pH 7.6), 100 mM potassium glutamate, 5 mM MgCl_2_, and 0.4 mM DTT) and incubated with 40x excess monomeric streptavidin (3) for 10 minutes prior to incubation with Cas9/dCas9.

*DNA Tracing and Refinement with Sub-Pixel Resolution*

Acquired AFM images were flattened, then leveled plane-wise, by line, and by 3rd order polynomial using an open-source image analysis software for scanning probe microscopy, Gwyddion (http://gwyddion.net/), then exported to MATLAB (Mathworks, Inc.). 151 x 151 pixel (405 nm x 405 nm) regions containing each DNA molecule were sorted by inspection for a clearly identifiable streptavidin label, the presence of at least one bound Cas9/dCas9 molecule, and an unambiguous end-to-end path (in particular, for lack of aggregation or overlap with other DNA molecules). The contour of the DNA was traced by hand and the estimated boundaries of the streptavidin and Cas9/dCas9 were marked. The trace was refined using a method inspired by Wiggins *et al*. (4).

Starting at the weighted centroid of the streptavidin (**x_1_**), the position of next element of the backbone (**x_2_**) is estimated by stepping 2.5 nm toward the nearest hand-traced points beyond the estimated boundary of the streptavidin. An 11-pixel line is drawn on a two-fold linear interpolation of the image of the DNA perpendicularly to the (**x_1_**-**x_2_**) line segment at **x_2_**. **x_2_** is relocated to the position on the normal line with the maximum topographical height then adjusted to the 2.5 nm from **x_1_** on the new (**x_1_**-**x_2_**) line. The positions of **x_3_** … **x_n_** are then iteratively estimated using the nearest hand-traced points to generate the initial guess for the next backbone position then corrected as before, and the correction process continues until the point **x_n_** is less than 2.5 nm from the end of the traced DNA molecule. When the refined trace enters the estimated boundary of a Cas9/dCas9 molecule at **x_i_**, the position of the DNA is instead estimated as the point on a cubic Hermite spline (using points **x_i-1_**, **x_i_**, **x_j_**, and **x_j+1_**, where **x_j_** is the first point of the hand-drawn trace beyond the estimated Cas9/dCas9 boundary) located 2.5 nm from **x_i_**.

Upon completion of the trace, the height of the DNA along the contour is extracted (relative to the median pixel height of the local region). The estimated boundaries of the streptavidin and Cas9/dCas9 were iteratively expanded or retracted around the original estimate until they expanded to a contiguous region greater than (μ_d_ + σ_d_), where μ_d_ and σ_d_ are the mean and standard deviation of the height of the traced DNA beyond the estimated positions of bound proteins, and the estimate converges.

To account for any instrumental hysteresis which may distort the apparent length of DNA, the length of the DNA was normalized, and only DNA molecules originally measured to be 20% of their expected length (given the known number of base-pairs, 0.33 nm per base-pair) were used for further analysis (for the AAVS1 substrate- number traced: 804; nominal length: 1198 bp, mean length recorded: 1283 bp, std. dev: 154 bp; for the engineered substrate- number traced: 1520, nominal length: 986 bp, mean length recorded: 1071 bp, std. dev: 124 bp; for the ‘nonsense’ substrate- number traced: 616, nominal length: 1078 bp, mean length recorded: 1217 bp, std. dev: 135 bp). This step prevented us from improperly analyzing, *e.g.*, two DNA molecules which appeared collinear, DNA which may have fragmented, or DNA which may have been cleaved by Cas9 and separated (which was rare, see main text).

*Kinetic Monte Carlo (KMC) of Guide RNA Strand Invasion and R-loop ‘Breathing’*

Kinetic Monte Carlo (KMC) experiments to simulate strand invasion by the guide RNAs at protospacer sites were performed using a Gillespie-type (5) (continuous time, discrete state) algorithm implemented in MATLAB. Strand invasion is modeled as a one-dimensional random walk in a position-dependent potential determined by the relative the nearest-neighbor dependent DNA:DNA and RNA:DNA binding free energies. That is, the guide RNA is base-paired with the protospacer up to protospacer site *m* (1 ≥ *m* ≥ 20 for sgRNA and 1 ≥ *m* ≥ 18 for a truncated sgRNA (tru-gRNA)) and, to first-order, the forward rate (rate of additional guide RNA invasion) *v_f_* is estimated using the symmetric approximation to be exp(-(ΔG°(*m* +1)_RNA:DNA_ – ΔG°(*m* +1)_DNA:DNA_)/2RT), where R is Boltzmann’s constant, T is the temperature (here 37°C to correspond with parameter set we used), ΔG°(*m* + 1)_RNA:DNA_ is free energy of the base-pairing between the RNA and protospacer at site *m* + 1 and ΔG°(*m* + 1)_DNA:DNA_ is the free energy of the base-pairing between the protospacer and its complementary DNA strand (the 1/2 corrective term is included to satisfy detailed balance). *v_f_* at state *m* = 20 or 18 for sgRNA or tru-gRNA was set to 0. The reverse rate (rate of re-hybridization between the protospacer and its complementary DNA strand) *v_r_* is calculated similarly as proportional to exp(-(ΔG°(*m*)_DNA:DNA_ - ΔG°(*m*)_RNA:DNA_)/2RT); if state *m* = 1, the simulation was halted (signifying guide RNA – protospacer dissociation). Starting at time *t* = 0 (in arbitrary time units), for each iteration of the algorithm, the *m*-dependent rates are determined and two random numbers *r_1_* and *r_2_* are generated from a uniform distribution between 0 and 1. *t* is advanced by Δ*t* = log(*r_1_*)/(*v_f_* + *v_r_*). State *m* is increased to *m* + 1 if *r_2_* ≥ *v_f_* /(*v_f_* + *v_r_*) or decreased to *m* – 1 otherwise. For ‘equilibrium’ measurements of R-loop breathing, *m* was initiated at *m* = 20 (or 18 in the case of tru-gRNA) and the algorithm iterated until *t* ≥ 10,000. For measurements of ‘invasion’ kinetics’ dynamics (such as in the presence of mismatched base-pairs), *m* was initiated at *m* = 10 (up to *t* = 1000).

Free energy parameters are derived from the literature from experiments at 1M NaCl at 37°C— sequence-dependent free energies ΔG°(x)_DNA:DNA_ were obtained from Ref. (6)_;_ ΔG°(x)_RNA:DNA_ were obtained from Ref. (7); and ΔG°(x)_RNA:DNA_ values in cases of introduced point mismatches rG·dG, rC·dC, rA·dA, and rU·dT were obtained from Ref. (8) (under slightly higher salt conditions). The sequence of the protospacer used is ‘ATCCTGTCCCTAGTGGCCCC’, the AAVS1 target site as in the AFM experiments; the sequence of the protospacer complementary DNA is ‘GGGGCCACTAGGGACAGGAT’, and the sequence of the guide RNA was either ‘GGGGCCACUAGGGACAGGAU’ for sgRNA or ‘GGCCACUAGGGACAGGAU’ for the truncated RNA.

*Correlations between R-loop stability derived from KMC and experimental Cas9 cleavage rates*

To analyze correlations between guide RNA – protospacer interactions and Cas9 cleavage rates *in vivo*, we extracted the sequences of guide RNAs and targeted DNA from Hsu *et al*. (9) and their experimentally determined maximum likelihood estimate (MLE) cutting frequencies by Cas9. The sequences for experiments with single-nucleotide PAM-distal (>10 bp away from the PAM site) rG·dG, rC·dC, rA·dA, and rU·dT mismatches were imported (*n* = 136) into the KMC script and simulations of strand invasion starting at *m* = 10 were repeated 1000 times for each sequence (up to *t* = 100) to obtain the mean fraction of *m* ≥ 16 occupancy times. Significance was determined by bootstrapping the mean fraction of occupancy with the MLE cutting frequencies *via* permutation 100,000 times, then recalculating correlation coefficients and *p*-values. Guide RNA – protospacer binding free energies were estimated by summing over the nearest neighbor energies using the parameter sets listed above and corrected with a -3.1 kcal mol^-1^ initiation factor.

***Supplementary Comment 1:* Correction of dCas9-tru-gRNA and dCas9-hp-gRNA data for comparison with dCas9-sgRNA structural properties**

We note that when comparing height and volume measurements of the proteins across experiments, it is important the AFM imaging conditions remain mostly consistent so as not to introduce artefacts (10). This does not generally present an issue, for example, when comparing heights and volumes of dCas9 bound to different sites on the engineered DNA molecules, but presents a challenge when comparing the structural properties of dCas9/Cas9 when using different guide RNAs or DNA substrates. As a control, we recorded the heights and volumes of the streptavidin proteins used to label the ends of the traced DNA molecules, which should remain unchanged across all experimental conditions, for the different experiments. For experiments with sgRNAs, mean heights of the streptavidins differed by less than 0.1 nm (mean difference: 0.087 nm; standard deviation of differences: 0.052 nm) and their mean volumes (1098 nm^3^) differed by less than 15 nm^3^ (mean difference: 14.461nm^3^; standard deviation of differences: 10.419 nm^3^). However, the mean heights and volumes between the experiments with tru-gRNA and the hp-gRNAs differed from those with sgRNAs by up to 0.14 nm and 225 nm^3^, respectively. So that we could directly compare the results of these experiments, the heights of dCas9 with tru-gRNA and hp-gRNAs on engineered DNA were shifted by their difference in mean heights relative to those with sgRNAs and the volumes scaled by the percent difference of the mean volumes.


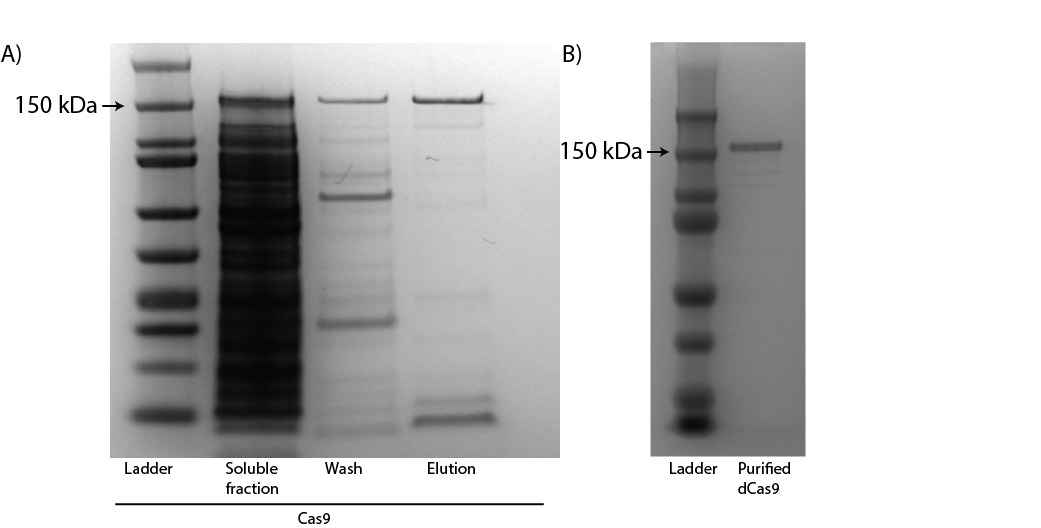


**Figure S1.** SDS gel of purified (A) Cas9 and (B) dCas9 products (nominal molecular weight: 160 kDa). Eluted bands show product is ~95% pure.


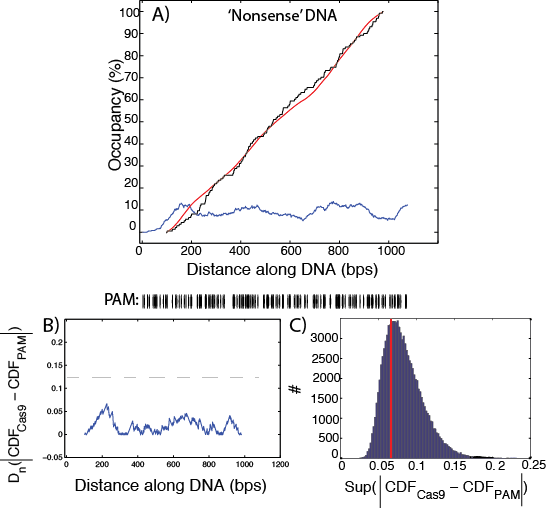


**Figure S2:** A) Binding distribution of dCas9 to substrate containing no homology to the AAVs1 protospacer sequence (compare with Figure 1) (n = 443). Overlaid is the cumulative distribution (CDF) of PAM sites (CDF_PAM_, black) and CDF of bases bound by dCas9 (red, CDF_Cas9_). Comparison begins 100 bases from each end to avoid artifacts introduced by overlap with streptavidin tag (a criteria for DNA selection) and binding to exposed blunt ends of DNA (resulting in expected increase in non-specific binding (11)). B) Absolute difference D_n_ between CDF of protein binding and of PAM sites. Dashed line is Kolmogorov-Smirnov criterion for goodness-of-fit of two distributions. C) CDF of binding was compared to CDF of PAM distributions from 100,000 randomly generated sequences with same probabilities of G, A, T, and C using MATLAB. Vertical red line is experimental Sup(D_n_), indicating that experimental dCas9 binding more closely matches the experimental PAM distribution than it does to 71.20% of generated sequences.

*
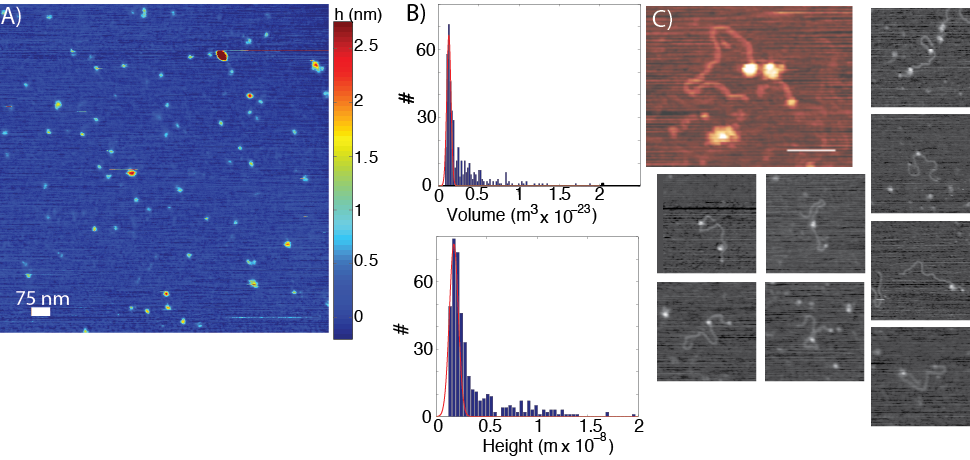
*

**Figure S3.** (A) Images of dCas9 alone. (B) Histogram (*n* = 423) of volume (left) and height (right) of dCas9 imaged alone with Gaussian fit to primary peaks. From the Gaussian fits: mean height is 1.746 nm (95% confidence: 1.689 nm – 1.802 nm) with standard deviation 0.441 nm, and mean volume is 1302 nm^3^ (95% confidence: 1266 nm^3^ – 1337 nm^3^) with standard deviation 259.1 nm^3^ (note that because the dCas9 here do not have a DNA within its binding channel, their recorded volumes may appear artificially low because of decreased mechanical resistance to the AFM probe). The heights were measured relative to the median value of a 10-pixel area surrounding each protein, and the volumes recorded as the contiguous features greater than twice the standard deviation of the local background heights. (C) Additional representative images of dCas9 bound to DNA which has been labeled at one end with a monovalent streptavidin.

*
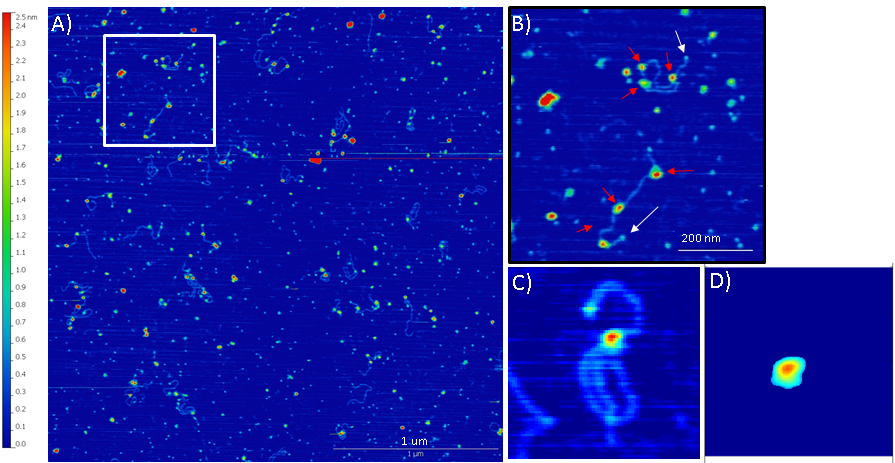
*

**Figure S4:** (A) Representative wide-field image of dCas9 bound to engineered DNA. (B) Close-up of boxed region. White arrows are monovalent streptavidin and red arrows are dCas9 proteins. C-D) Example of extraction from original image (C) and isolation (D) of Cas9/dCas9 structures (see Online Methods). This extraction was repeated for each isolated protein bound to the DNA, then aligned pair-wise through iterative translation, rotation, and reflection to minimize their mean-squared topological difference. From these minimized mean-squared differences we composed a distance matrix, clustered each protein according to the method of Laio and Rodriguez (12), then mapped the populations of structures by cluster back to their sites on the DNA (Figure 2A, Figure S3).

*
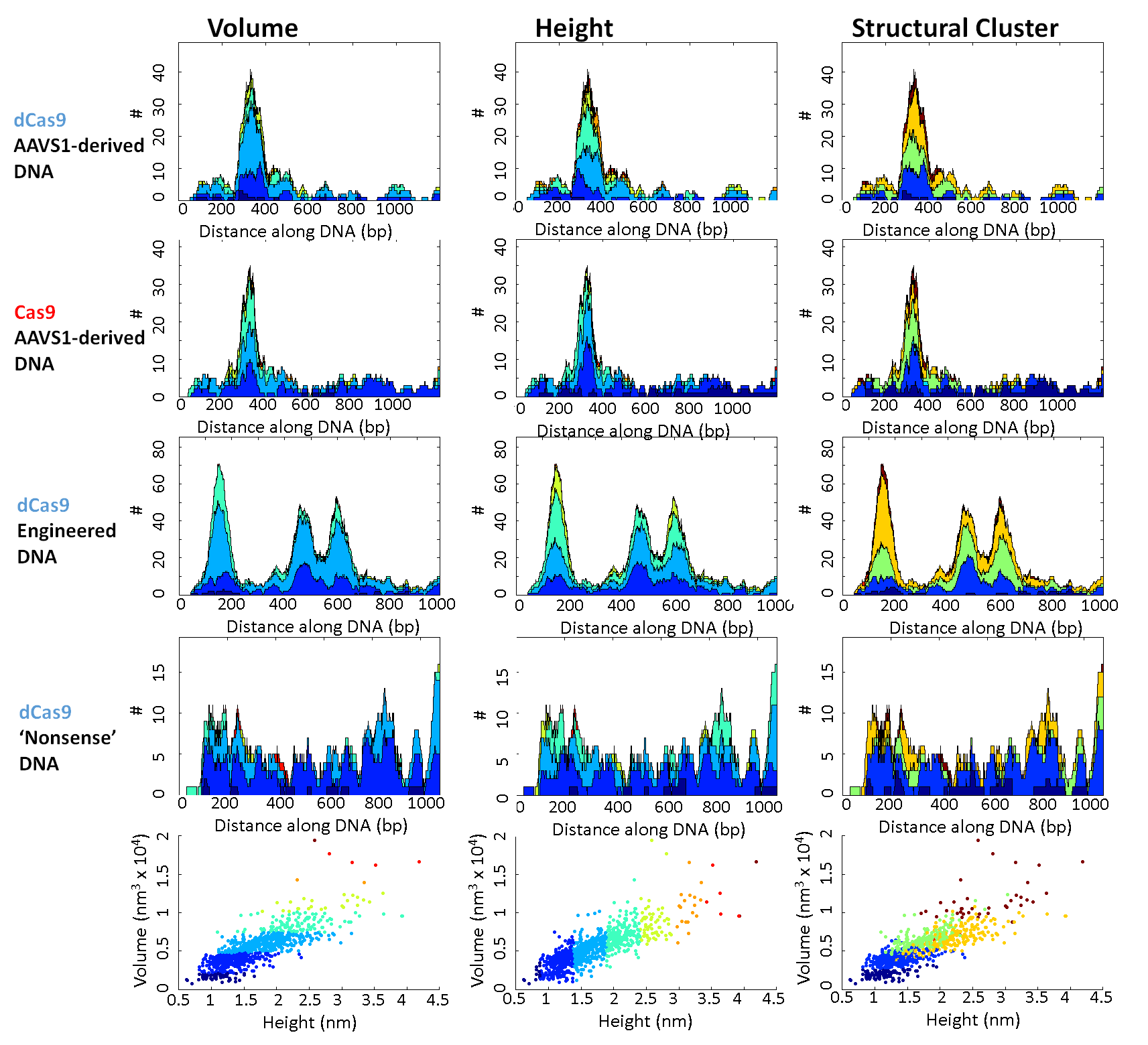
*

**Figure S5:** Upper: ***Stacked*** histograms of the volume (left), maximum heights (middle), and structures (clustered by mean squared difference) after alignment (right, see text) for all experimental conditions. Populations are colored according to binned volume, height or structural cluster as in the scatter plot below. The binding distribution of extracted Cas9/dCas9 molecules (Figure S3) closely matches that of the entire dataset (Figure 1C-D, Figure S1), indicating that the selection procedure is unbiased and the selected proteins are representative of the whole data set. Lower: Scatter plot of volume vs. maximum height of all Cas9/dCas9 color-coded by binned (left) volume, (middle) maximum height, and (right) structural cluster.


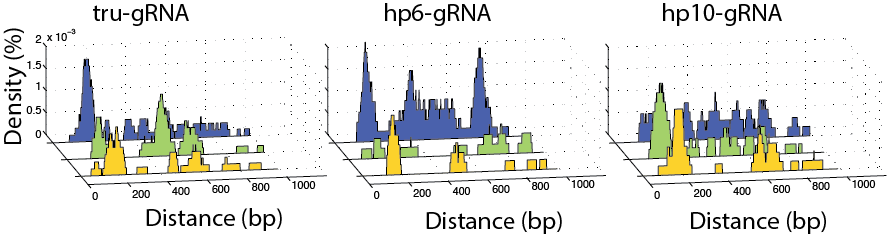


**Figure S6.** Fraction of bound DNA occupied by Cas9/dCas9 with along the engineered DNA substrate, with colors representing populations of Cas9/dCas9 clustered according to their structures (see Figure 3C in main text). Protein structures were classified according to the dCas9/Cas9 with sgRNA that they most closely resembled (by mean-squared difference after alignment, see text). For reference, on the engineered DNA substrates, location of full protospacer site: 144 – 167 bp; location of 10 MM (8MM) site: 452 – 465 bp; location of 5MM (3MM) site: 592 – 610 bp.

Here we see similar trends as with dCas9/Cas9 with sgRNAs: as dCas9 binds to sites which increasingly match the mismatch, the fraction of population clustering with the largest (yellow) group increases, although this effect is depressed in tru-gRNA, with a sizable fraction of the population clustering with smaller (green and blue) populations even at the full protospacer site. The effect for hp10-gRNA is particularly pronounced, emphasizing that it has poor affinities for off-target sites.

**
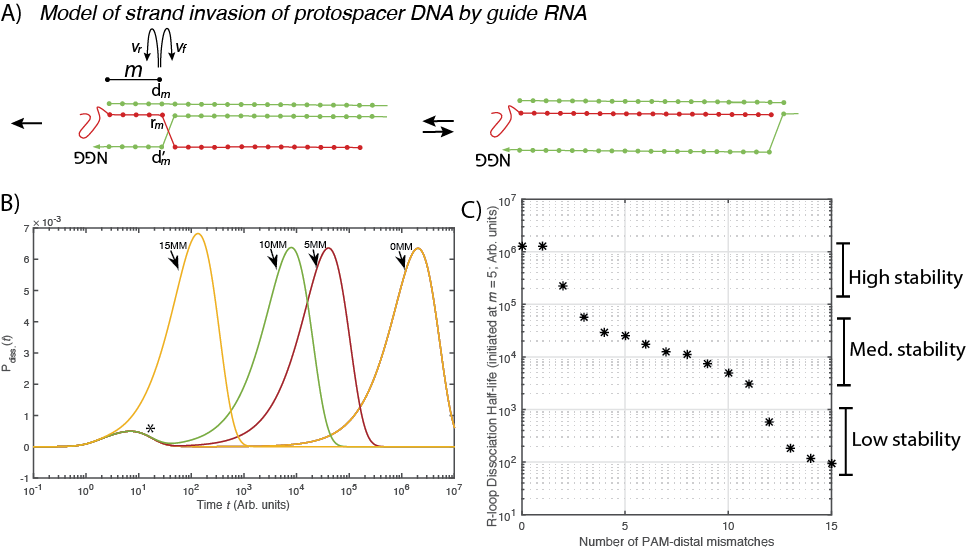
**

**Figure S7.** (A) Schematic model of strand invasion of DNA protospacers by guide RNAs. See also Figure 4A in the main text and Supplementary Methods. Guide RNA is presumed to dissociated when *m* = 1. (B) Calculated probability distribution of dissociation times for a guide RNA initially invaded up to *m* = 5 for protospacers with different numbers of contiguous PAM-distal mismatches. The length of these dissociation times can be viewed as an approximation of dCas9 binding propensity at those sites. The asterisk highlights the dissociation times for the population of guide RNAs which initially fails to fully invade after initial invasion to *m* = 5. The invaded RNAs are highly unstable at protospacer sites with 15 PAM-distal mismatches (15MM), and experimentally we rarely observe Cas9/dCas9 bound at these sites (Figure 1D). The invaded RNA (prior to dissociation) at protospacer sites with 10 or 5 PAM-distal mismatches (10MM and 5MM) are calculated to remain for significantly longer than those at 15MM sites, but within an order of magnitude of each other; we find their binding propensity to be approximately equal and lower than full protospacer sites (0MM) in AFM experiments. The probability density functions were calculated using a Q-matrix method as described in Ref. (13), using the sequence-specific transition rates between the *m* states (*v_f_* and *v_r_*, see Supplementary Methods). (C) Examination of the estimated half-lives of RNA-protospacer binding at protospacers with different numbers of PAM-distal mismatches suggests there are roughly three regimes within which the stabilities of the invaded RNA are similar: those with > 11 PAM-distal mismatches (low stability); those with between 3 and 11 PAM-distal mismatches (medium stability); and those with < 3 PAM-distal mismatches (high stability). The results are qualitatively similar to the distribution of dCas9 on the engineered substrate observed *via* AFM (Figure 1D) as well as those reported elsewhere (14).


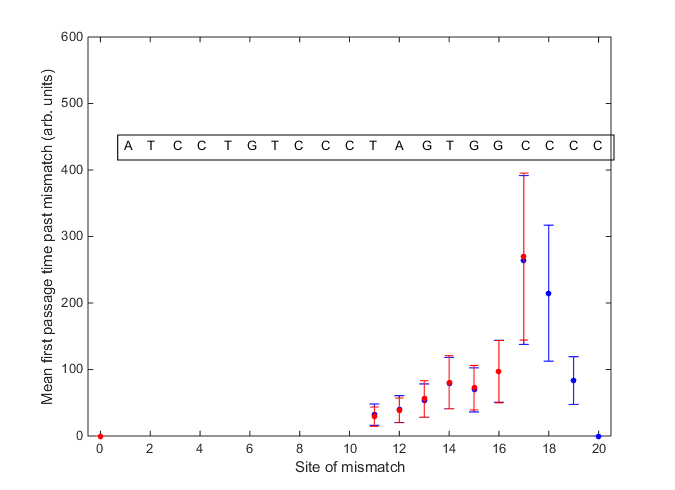


**Figure S8.** Simulated (kinetic Monte Carlo) mean first passage times to traverse the mismatched site during strand invasion by sgRNA (blue) and tru-gRNA (red) for different positions of the mismatched site. Error bars are standard deviations of recorded first passage times. Sequence of protospacer (AAVS1 site) in box.


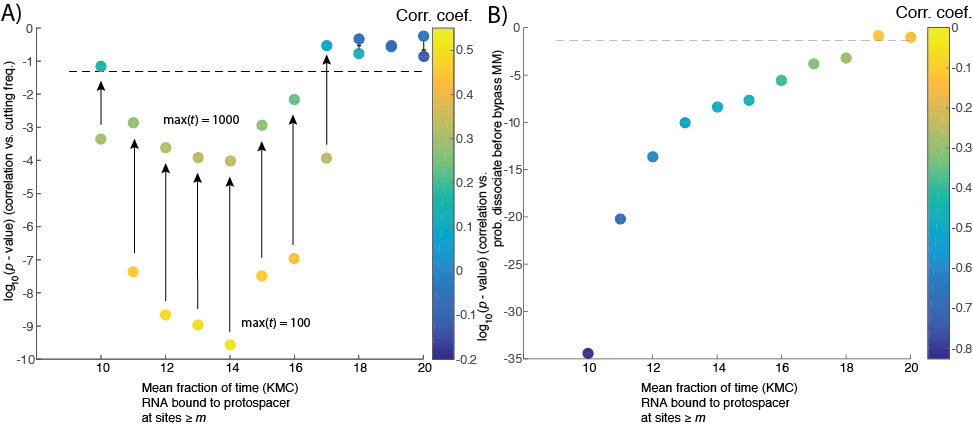


**Figure S9.** (A) Statistical power and strength of the correlations between stability of R-loop sites (from kinetic Monte Carlo, see main text) and experimental cleavage frequency from Hsu *et al.* (9) decrease with increasing simulation length (max(*t*) = 100 to max(*t*) = 1000, arbitrary units). This result suggests that the kinetics of strand invasion can be an important predictor of off-target cleavage rate. (B) Correlation between fractions of time the R-loop is of size *m* *vs.* the probability that the kinetic Monte Carlo trial predicts that the invading strand will dissociate before traversing the mismatch. Binding at sites 10 ~ 14-15 is very strongly anti-correlated (~0.5-0.85) with the probability of dissociation before traversing the mismatch, while from the AFM imaging experiments we find that binding at sites ~≥16 are associated with a conformational change in the Cas9/dCas9.

**Table S1. Sequences and Maximum Likelihood Estimate (MLE) Cutting Frequencies from Hsu *et al*. (2013) (9) used for correlation analysis** (mismatch site in target sequence bold).

Only those sequences with MLE cutting frequency and with a single, isolated, PAM-distal (≥ 10 sites from PAM) mismatch of type rG·dG, rC·dC, rA·dA, or rU·dT were analyzed from the data set.

| **Target sequence** | **Protospacer-targeting region of Guide RNA** | **MLE Cutting Frequency**  **(Hsu *et al*. (2013) (9))** | **Estimated ΔG°_37_ (kcal/mol)** |
| --- | --- | --- | --- |
| TTCTTCTTCTGCTCGGAC**T**C | GUGUCCGAGCAGAAGAAGAA | 0.10384 | -32.16 |
| TTCTTCTTCTGCTCGGA**C**TC | GACUCCGAGCAGAAGAAGAA | 0.12609 | -31.4 |
| TTCTTCTTCTGCTCGG**A**CTC | GAGACCGAGCAGAAGAAGAA | 0.13145 | -32.69 |
| TTCTTCTTCTGCTCG**G**ACTC | GAGUGCGAGCAGAAGAAGAA | 0.097464 | -32.33 |
| TTCTTCTTCTGCTC**G**GACTC | GAGUCGGAGCAGAAGAAGAA | 0.12704 | -33.43 |
| TTCTTCTTCTGCT**C**GGACTC | GAGUCCCAGCAGAAGAAGAA | 0.079556 | -31.37 |
| TTCTTCTTCTGC**T**CGGACTC | GAGUCCGUGCAGAAGAAGAA | 0.11197 | -32.36 |
| TTCTTCTTCTG**C**TCGGACTC | GAGUCCGACCAGAAGAAGAA | 0.04788 | -31.9 |
| TTCTTCTTCT**G**CTCGGACTC | GAGUCCGAGGAGAAGAAGAA | 0.085461 | -32.83 |
| TTCTTCTTC**T**GCTCGGACTC | GAGUCCGAGCUGAAGAAGAA | 0.074938 | -32.22 |
| TTCTTCTTCTGCTCGGAC**T**C | GUGUCCGAGCAGAAGAAGAA | 0.15588 | -32.16 |
| TTCTTCTTCTGCTCGGA**C**TC | GACUCCGAGCAGAAGAAGAA | 0.11015 | -31.4 |
| TTCTTCTTCTGCTCGG**A**CTC | GAGACCGAGCAGAAGAAGAA | 0.11435 | -32.69 |
| TTCTTCTTCTGCTCG**G**ACTC | GAGUGCGAGCAGAAGAAGAA | 0.15072 | -32.33 |
| TTCTTCTTCTGCTC**G**GACTC | GAGUCGGAGCAGAAGAAGAA | 0.11567 | -33.43 |
| TTCTTCTTCTGCT**C**GGACTC | GAGUCCCAGCAGAAGAAGAA | 0.070181 | -31.37 |
| TTCTTCTTCTGC**T**CGGACTC | GAGUCCGUGCAGAAGAAGAA | 0.10538 | -32.36 |
| TTCTTCTTCTG**C**TCGGACTC | GAGUCCGACCAGAAGAAGAA | 0.064145 | -31.9 |
| TTCTTCTTCT**G**CTCGGACTC | GAGUCCGAGGAGAAGAAGAA | 0.085148 | -32.83 |
| TTCTTCTTC**T**GCTCGGACTC | GAGUCCGAGCUGAAGAAGAA | 0.064903 | -32.22 |
| CCCTAGTCATTGGAGGTG**A**C | GACACCUCCAAUGACUAGGG | 0.062949 | -32.19 |
| CCCTAGTCATTGGAGGT**G**AC | GUGACCUCCAAUGACUAGGG | 0.063313 | -31.73 |
| CCCTAGTCATTGGAGG**T**GAC | GUCUCCUCCAAUGACUAGGG | 0.068655 | -31.72 |
| CCCTAGTCATTGGAG**G**TGAC | GUCAGCUCCAAUGACUAGGG | 0.073003 | -32 |
| CCCTAGTCATTGGA**G**GTGAC | GUCACGUCCAAUGACUAGGG | 0.037401 | -32.63 |
| CCCTAGTCATTGG**A**GGTGAC | GUCACCACCAAUGACUAGGG | 0.038197 | -32.11 |
| CCCTAGTCATTG**G**AGGTGAC | GUCACCUGCAAUGACUAGGG | 0.041758 | -31.63 |
| CCCTAGTCATT**G**GAGGTGAC | GUCACCUCGAAUGACUAGGG | 0.067751 | -32.23 |
| CCCTAGTCAT**T**GGAGGTGAC | GUCACCUCCUAUGACUAGGG | 0.031653 | -31.62 |
| CCCTAGTCA**T**TGGAGGTGAC | GUCACCUCCAUUGACUAGGG | 0.027161 | -31.77 |
| ATGGGGAGGACATCGATG**T**C | GUCAUCGAUGUCCUCCCCAU | 0.027124 | -31.26 |
| ATGGGGAGGACATCGAT**G**TC | GAGAUCGAUGUCCUCCCCAU | 0.022366 | -31.7 |
| ATGGGGAGGACATCGA**T**GTC | GACUUCGAUGUCCUCCCCAU | 0.01127 | -30.92 |
| ATGGGGAGGACATCG**A**TGTC | GACAACGAUGUCCUCCCCAU | 0.011836 | -31.44 |
| ATGGGGAGGACATC**G**ATGTC | GACAUGGAUGUCCUCCCCAU | 0.009146 | -31.83 |
| ATGGGGAGGACAT**C**GATGTC | GACAUCCAUGUCCUCCCCAU | 0.006333 | -30.27 |
| ATGGGGAGGACA**T**CGATGTC | GACAUCGUUGUCCUCCCCAU | 0.006232 | -31.06 |
| ATGGGGAGGAC**A**TCGATGTC | GACAUCGAAGUCCUCCCCAU | 0.007085 | -31.64 |
| ATGGGGAGGA**C**ATCGATGTC | GACAUCGAUCUCCUCCCCAU | 0.001545 | -30.32 |
| ATGGGGAGG**A**CATCGATGTC | GACAUCGAUGACCUCCCCAU | 0.00025 | -31.59 |
| ATCACATCAACCGGTGGC**G**C | GGGCCACCGGUUGAUGUGAU | 0.15963 | -35.23 |
| ATCACATCAACCGGTGG**C**GC | GCCCCACCGGUUGAUGUGAU | 0.14121 | -32.17 |
| ATCACATCAACCGGTG**G**CGC | GCGGCACCGGUUGAUGUGAU | 0.18743 | -33.43 |
| ATCACATCAACCGGT**G**GCGC | GCGCGACCGGUUGAUGUGAU | 0.1634 | -33.63 |
| ATCACATCAACCGG**T**GGCGC | GCGCCUCCGGUUGAUGUGAU | 0.15877 | -33.12 |
| ATCACATCAACCG**G**TGGCGC | GCGCCAGCGGUUGAUGUGAU | 0.029249 | -33.4 |
| ATCACATCAACC**G**GTGGCGC | GCGCCACGGGUUGAUGUGAU | 0.12208 | -34.13 |
| ATCACATCAAC**C**GGTGGCGC | GCGCCACCCGUUGAUGUGAU | 0.051622 | -31.57 |
| ATCACATCAA**C**CGGTGGCGC | GCGCCACCGCUUGAUGUGAU | 0.004914 | -31.74 |
| ATCACATCA**A**CCGGTGGCGC | GCGCCACCGGAUGAUGUGAU | 0.032227 | -33.79 |
| GAGTTTCTCATCTGTG**C**CCC | GGGCCACAGAUGAGAAACUC | 0.015879 | -33.54 |
| CCAGCTTCTGCCGTTTG**T**AC | GUUCAAACGGCAGAAGCUGG | 0.037469 | -33.17 |
| CCAGCTTCTGCCGTT**T**GTAC | GUACUAACGGCAGAAGCUGG | 0.059921 | -32.92 |
| CCAGCTTCT**G**CCGTTTGTAC | GUACAAACGGGAGAAGCUGG | 0.032605 | -33.43 |
| TTCCTCCTCCAGCTTCTG**C**C | GCCAGAAGCUGGAGGAGGAA | 0.000481 | -35.94 |
| TTCCTCCTCCAGCTT**C**TGCC | GGCACAAGCUGGAGGAGGAA | 0.041538 | -37.4 |
| TTCCTCCTCCAG**C**TTCTGCC | GGCAGAACCUGGAGGAGGAA | 0.047874 | -37.5 |
| TTCCTCCTCC**A**GCTTCTGCC | GGCAGAAGCAGGAGGAGGAA | 0.050381 | -38.61 |
| TTCCTCCTC**C**AGCTTCTGCC | GGCAGAAGCUCGAGGAGGAA | 0.006459 | -36.92 |
| CCGGTTGATGTGATGGG**A**GC | GCACCCAUCACAUCAACCGG | 0.03967 | -33.31 |
| CCGGTTGATGTGA**T**GGGAGC | GCUCCCUUCACAUCAACCGG | 0.033426 | -32.52 |
| CCGGTTGATGTG**A**TGGGAGC | GCUCCCAACACAUCAACCGG | 0.035651 | -33.04 |
| CCGGTTGAT**G**TGATGGGAGC | GCUCCCAUCAGAUCAACCGG | 0.03209 | -33.3 |
| GCAGCAAGCAGCAC**T**CTGCC | GGCAGUGUGCUGCUUGCUGC | 0.004014 | -32.46 |
| GCAGCAAGC**A**GCACTCTGCC | GGCAGAGUGCAGCUUGCUGC | 0.000219 | -33.11 |
| GCTTGGGCCCACGC**A**GGGGC | GCCCCAGCGUGGGCCCAAGC | 0.001487 | -38.81 |
| GCTTGGGCCCA**C**GCAGGGGC | GCCCCUGCCUGGGCCCAAGC | 0.003322 | -36.77 |
| GCTTCGTGGCAATG**C**GCCAC | GUGGCCCAUUGCCACGAAGC | 0.000463 | -32.67 |
| GCTTGGGCC**C**ACGCAGGGGC | GCCCCUGCGUCGGCCCAAGC | 0 | -37.12 |
| AAGCTGGACTC**T**GGCCACTC | GAGUGGCCUGAGUCCAGCUU | 0.010169 | -33.02 |
| TTCTTCTTCTGCTCGG**A**CTC | GAGACCGAGCAGAAGAAGAA | 0.084395 | -32.69 |
| TTCTTCTTCT**G**CTCGGACTC | GAGUCCGAGGAGAAGAAGAA | 0.051852 | -32.83 |
| TTCTTCTTC**T**GCTCGGACTC | GAGUCCGAGCUGAAGAAGAA | 0.050685 | -32.22 |
| GAGTTTCTCA**T**CTGTGCCCC | GGGGCACAGUUGAGAAACUC | 0.004503 | -34.16 |
| TTCCTCCTCCA**G**CTTCTGCC | GGCAGAAGGUGGAGGAGGAA | 0.006035 | -38.83 |
| TTCCTCCTCC**A**GCTTCTGCC | GGCAGAAGCAGGAGGAGGAA | 0.011364 | -38.61 |
| AGCAGAAGAA**G**AAGGGCTCC | GGAGCCCUUGUUCUUCUGCU | 0.007206 | -29.83 |
| AAGCTGGACTC**T**GGCCACTC | GAGUGGCCUGAGUCCAGCUU | 0 | -33.02 |
| CCCTAGTCATTGGAGGTG**A**C | GACACCUCCAAUGACUAGGG | 0.053611 | -32.19 |
| CCCTAGTCATTGGAGGT**G**AC | GUGACCUCCAAUGACUAGGG | 0.05399 | -31.73 |
| CCCTAGTCATTGGAGG**T**GAC | GUCUCCUCCAAUGACUAGGG | 0.070404 | -31.72 |
| CCCTAGTCATTGGAG**G**TGAC | GUCAGCUCCAAUGACUAGGG | 0.067678 | -32 |
| CCCTAGTCATTGGA**G**GTGAC | GUCACGUCCAAUGACUAGGG | 0.03597 | -32.63 |
| CCCTAGTCATTGG**A**GGTGAC | GUCACCACCAAUGACUAGGG | 0.025207 | -32.11 |
| CCCTAGTCATTG**G**AGGTGAC | GUCACCUGCAAUGACUAGGG | 0.056019 | -31.63 |
| CCCTAGTCATT**G**GAGGTGAC | GUCACCUCGAAUGACUAGGG | 0.065347 | -32.23 |
| CCCTAGTCAT**T**GGAGGTGAC | GUCACCUCCUAUGACUAGGG | 0.063769 | -31.62 |
| CCCTAGTCA**T**TGGAGGTGAC | GUCACCUCCAUUGACUAGGG | 0.052644 | -31.77 |
| ATGGGGAGGACATCGATG**T**C | GUCAUCGAUGUCCUCCCCAU | 0.020295 | -31.26 |
| ATGGGGAGGACATCGAT**G**TC | GAGAUCGAUGUCCUCCCCAU | 0.012126 | -31.7 |
| ATGGGGAGGACATCGA**T**GTC | GACUUCGAUGUCCUCCCCAU | 0.007202 | -30.92 |
| ATGGGGAGGACATCG**A**TGTC | GACAACGAUGUCCUCCCCAU | 0.010912 | -31.44 |
| ATGGGGAGGACATC**G**ATGTC | GACAUGGAUGUCCUCCCCAU | 0.009292 | -31.83 |
| ATGGGGAGGACAT**C**GATGTC | GACAUCCAUGUCCUCCCCAU | 0.006125 | -30.27 |
| ATGGGGAGGACA**T**CGATGTC | GACAUCGUUGUCCUCCCCAU | 0.007805 | -31.06 |
| ATGGGGAGGAC**A**TCGATGTC | GACAUCGAAGUCCUCCCCAU | 0.010174 | -31.64 |
| ATGGGGAGGA**C**ATCGATGTC | GACAUCGAUCUCCUCCCCAU | 0.003595 | -30.32 |
| ATGGGGAGG**A**CATCGATGTC | GACAUCGAUGACCUCCCCAU | 0.000206 | -31.59 |
| ATCACATCAACCGGTGGC**G**C | GGGCCACCGGUUGAUGUGAU | 0.18977 | -35.23 |
| ATCACATCAACCGGTGG**C**GC | GCCCCACCGGUUGAUGUGAU | 0.13525 | -32.17 |
| ATCACATCAACCGGTG**G**CGC | GCGGCACCGGUUGAUGUGAU | 0.14749 | -33.43 |
| ATCACATCAACCGGT**G**GCGC | GCGCGACCGGUUGAUGUGAU | 0.13952 | -33.63 |
| ATCACATCAACCGG**T**GGCGC | GCGCCUCCGGUUGAUGUGAU | 0.13949 | -33.12 |
| ATCACATCAACCG**G**TGGCGC | GCGCCAGCGGUUGAUGUGAU | 0.031221 | -33.4 |
| ATCACATCAACC**G**GTGGCGC | GCGCCACGGGUUGAUGUGAU | 0.14776 | -34.13 |
| ATCACATCAAC**C**GGTGGCGC | GCGCCACCCGUUGAUGUGAU | 0.050539 | -31.57 |
| ATCACATCAA**C**CGGTGGCGC | GCGCCACCGCUUGAUGUGAU | 0.003982 | -31.74 |
| ATCACATCA**A**CCGGTGGCGC | GCGCCACCGGAUGAUGUGAU | 0.015494 | -33.79 |
| GAGTTTCTCATCTGTG**C**CCC | GGGCCACAGAUGAGAAACUC | 0.025334 | -33.54 |
| CCAGCTTCTGCCGTTTG**T**AC | GUUCAAACGGCAGAAGCUGG | 0.062094 | -33.17 |
| CCAGCTTCTGCCGTT**T**GTAC | GUACUAACGGCAGAAGCUGG | 0.080429 | -32.92 |
| CCAGCTTCT**G**CCGTTTGTAC | GUACAAACGGGAGAAGCUGG | 0.032505 | -33.43 |
| TTCCTCCTCCAGCTTCTG**C**C | GCCAGAAGCUGGAGGAGGAA | 0.00117 | -35.94 |
| TTCCTCCTCCAGCTT**C**TGCC | GGCACAAGCUGGAGGAGGAA | 0.034381 | -37.4 |
| TTCCTCCTCCAG**C**TTCTGCC | GGCAGAACCUGGAGGAGGAA | 0.059128 | -37.5 |
| TTCCTCCTCC**A**GCTTCTGCC | GGCAGAAGCAGGAGGAGGAA | 0.05162 | -38.61 |
| TTCCTCCTC**C**AGCTTCTGCC | GGCAGAAGCUCGAGGAGGAA | 0.007682 | -36.92 |
| CCGGTTGATGTGATGGG**A**GC | GCACCCAUCACAUCAACCGG | 0.093725 | -33.31 |
| CCGGTTGATGTGA**T**GGGAGC | GCUCCCUUCACAUCAACCGG | 0.075435 | -32.52 |
| CCGGTTGATGTG**A**TGGGAGC | GCUCCCAACACAUCAACCGG | 0.091723 | -33.04 |
| CCGGTTGAT**G**TGATGGGAGC | GCUCCCAUCAGAUCAACCGG | 0.070319 | -33.3 |
| GCAGCAAGCAGCAC**T**CTGCC | GGCAGUGUGCUGCUUGCUGC | 0.006754 | -32.46 |
| GCAGCAAGC**A**GCACTCTGCC | GGCAGAGUGCAGCUUGCUGC | 0.000545 | -33.11 |
| GCTTGGGCCCACGC**A**GGGGC | GCCCCAGCGUGGGCCCAAGC | 0.004676 | -38.81 |
| GCTTGGGCCCA**C**GCAGGGGC | GCCCCUGCCUGGGCCCAAGC | 0.001918 | -36.77 |
| GCTTCGTGGCAATG**C**GCCAC | GUGGCCCAUUGCCACGAAGC | 0.001045 | -32.67 |
| GCTTGGGCC**C**ACGCAGGGGC | GCCCCUGCGUCGGCCCAAGC | 0 | -37.12 |
| AAGCTGGACTC**T**GGCCACTC | GAGUGGCCUGAGUCCAGCUU | 0.008891 | -33.02 |
| TTCTTCTTCTGCTCGG**A**CTC | GAGACCGAGCAGAAGAAGAA | 0.091861 | -32.69 |
| TTCTTCTTCT**G**CTCGGACTC | GAGUCCGAGGAGAAGAAGAA | 0.062783 | -32.83 |
| TTCTTCTTC**T**GCTCGGACTC | GAGUCCGAGCUGAAGAAGAA | 0.044444 | -32.22 |
| GAGTTTCTCA**T**CTGTGCCCC | GGGGCACAGUUGAGAAACUC | 0.0053 | -34.16 |
| TTCCTCCTCCA**G**CTTCTGCC | GGCAGAAGGUGGAGGAGGAA | 0.00714 | -38.83 |
| TTCCTCCTCC**A**GCTTCTGCC | GGCAGAAGCAGGAGGAGGAA | 0.019945 | -38.61 |
| AGCAGAAGAA**G**AAGGGCTCC | GGAGCCCUUGUUCUUCUGCU | 0.007996 | -29.83 |
| AAGCTGGACTC**T**GGCCACTC | GAGUGGCCUGAGUCCAGCUU | 0.006102 | -33.02 |

*DNA/RNA Sequences*

PAM sites labeled in **YELLOW** (for simplicity, only **GGT** PAM sites which match the PAM in the native AAVS1 target are labeled); sequences complementary to the protospacer labeled in **BLACK**; and sequences mismatched to protospacer sequences highlighted in **RED**.

*sgRNA:*

**GGGGCCACUAGGGACAGGAU**guuuuagagcuagaaauagcaaguuaaaauaaggcuaguccguuaucaacuugaaaaaguggcaccgagucggugcuuuu

*tru-gRNA:*

**GGCCACUAGGGACAGGAU**guuuuagagcuagaaauagcaaguuaaaauaaggcuaguccguuaucaacuugaaaaaguggcaccgagucggugcuuuu

*hp6-gRNA:*

gg**GGCCCC**uucg**GGGGCCACUAGGGACAGGAU**guuuuagagcuagaaauagcaaguuaaaauaaggcuaguccguuaucaacuugaaaaaguggcaccgagucggugcuuuu

*hp10-gRNA:*

gg**UAGUGGCCCC**uucg**GGGGCCACUAGGGACAGGAU**guuuuagagcuagaaauagcaaguuaaaauaaggcuaguccguuaucaacuugaaaaaguggcaccgagucggugcuuuu

*AAVS1-derived substrate:*

**CCA**ggatcagtgaaacgca**CCA**gacagccgcgtcagagcagctcaggttc**TGG**gagagggtagcgcaggg**TGGCCA**ctgagaaccgggcaggtcacgcatcccccccttccctc**CCA**ccccctg**CCA**agctctccctc**CCA**ggatcctctc**TGG**ct**CCA**tcgtaagcaaaccttagaggttc**TGG**caaggagagaga**TGG**ct**CCA**ggaaa**TGG**gggtgtgtca**CCA**gataaggaatctgcctaacaggagg**TGG**gggttagac**CCA**atatcaggagactaggaaggaggaggcctaagga**TGG**ggcttttctgtca**CCAATCCTGTCCCTAGTGGCCCC**actg**TGG**gg**TGG**aggggacagataaaagtac**CCA**gaa**CCA**gag**CCA**cattaaccggccc**TGG**gaatataagg**TGG**tc**CCA**gctcggggacacaggatccc**TGG**aggcagcaaacatgctgtcctgaag**TGG**acataggggcccgggt**TGG**aggaagaagactagctgagctctcggacccc**TGG**aagatg**CCA**tgacagggggc**TGG**aagagctagcacagactagagaggtaaggggggtaggggagctgc**CCA**aatgaaaggagtgagaggtgacccggaat**CCA**caggagaacggggtgt**CCA**ggcaaagaaagcaagagga**TGG**agagg**TGG**ctaaag**CCA**gggagacggggtactt**TGG**ggttgt**CCA**gaaaaacggtgatgatgcaggcctacaagaaggggaggcgggacgcaagggagacatccgtcggagaagg**CCA**tcctaagaaacgagaga**TGG**cacaggcc**CCA**gaaggagaaggaaaagggaac**CCA**gcgagtgaagacggca**TGG**ggt**TGG**gtgagggaggagagatgcccggagaggac**CCA**gacacggggaggatccgctcagaggacatcacg**TGG**tgcagcgccgagaaggaagtgctccggaaagagcatcct**TGG**gcagcaacacagcagagagcaaggggaagagggag**TGG**aggaagacggaacctgaaggaggcggcagggaaggatc**TGG**g**CCA**gccgtagaggtgac**CCA**gg**CCA**caagctgcagacagaaagcggcacaggc**CCA**ggggagagaatgcaggtcagagaaagcaggacctgcc**TGG**gaaggggaaacag**TGG**g**CCA**gaggcggcgcagaag**CCA**gtagagctc

*Engineered substrate:*

catgacgtgcagcaagcgcgctgacgcagctaattttatctatgtgcttcgtcatacgtgatgcatatactctctgctagctgactcattcagctgtactcactcgctgttgagtctcatacagcgcgagatcaaatgagtcat**CCAATCCTGTCCCTAGTGGCCCC**tatcgtgacactgcactgcagcgtacgcgacagagctagacttcgtttcaatacagactagctactgcgtctgcagagcgctctcttgtcacttacatcgaagtcaacgcgctcgcgttcagagatcttct**CCAATCCTACTTTCGACAATTTT**cgctctcagcgtttgtgtctgtgcgcgcacacagtctgtgcttcgcttgcaactaacgtagcgcttcagcgcatcgtcaaagagcgaaagagtcacagtgtctgtgttcacgtctctatctttctagttct**CCAATCCTGTCCCAAAATTGTCG**aagacagagtttcgcgaattcgcagcagcgcgatctctgctcactgcaatctctgactgctgcttttaagcaattctcgcagctcagcatgagtacttgcgattacagcagtgctcg**CCAATCCTGTCCCTAGTGATTTT**gcagcgtcagcagagctgatgagatgcagttcagcatcgcagacgagcagcacgaagcgagcatctgtagaaaatcgatcgacgcgcacttgcagagaca**CCAAAAATTGTCGAAAGTGAAGC**cttgtctctcgttcgcatcaagacacgctatttctgtctcttaaatgtttcaaaaacacatcatgtcttcttcgtgcgagttcgatgcgcgtgtgcgaga**CCACGCCTGTCCCTAGTGGCCCC**tgactctctgtgcattttgagctgcgaaagtaaagatatcgttcattgcagagacagagctagtactagtctcagttcttgcactatgctctcgatgtctctcttagtgattcagcgcatcgtcg

*“Nonsense” substrate:*

gacctgcaggcatgcaagct**TGG**gctagcggagagtcagttcgcggtac**TGG**aggaggcggcgcaacgtcg**CCA**gctgtctgcacaggagaaatccctgc**TGG**cgcataaagatgagacgc**TGG**agtacaaacg**CCA**gc**TGG**ctgcact**TGG**cgacaaggttacgtatcaggagcgcctgaacgcgc**TGG**cgcagcaggcggataaattcgcacagcagcaacgggcaaaacgggccg**CCA**ttgatgcgaaaagccgggggctgactgaccggcaggcagaacgggaag**CCA**cggaacagcgcctgaaggaacagta**TGG**cgataatccgc**TGG**cgctgaataacgtcatgtcagagcagaaaaagacc**TGG**gcggctgaaga**CCA**gcttcgcgggaac**TGG**a**TGG**caggcctgaagtccggc**TGGCCATGG**gctgagg**CCA**gctgaggtaccgctgaggattgctgaggtgtacagacgctcaagtcagagg**TGG**cgagagctcccggag**TGG**ctcacagtcgg**TGG**tccggcagtacaa**TGG**attaccgtaagacggaaatcactcccgggtatatgaaagagacga**CCA**ctg**CCA**gggacgaaagtgcaatgcggcatacctcag**TGG**cg**TGG**agtgcaggtatacagattaatccggcagcgtccgtcgttgttgatattgcttatgaaggctccggcag**TGG**cgac**TGG**cgtactgacggattcatcgt**TGG**ggtcggttataaattctgattag**CCA**ggtaacacagtgttatgacagcccgccggaaccgg**TGG**gcttttttg**TGG**ggtgaata**TGG**cagtaaagatttcaggagtcctgaaagacggcacaggaaaaccggtacagaactgca**CCA**ttcagctgaaag**CCA**gacgtaacagca**CCA**cgg**TGGTGG**tgaacacgg**TGG**gctcagagaatccggatgaagccgggcgttacagca**TGG**atg**TGG**agtacggtcagtacagtgtcatcctgcaggttgacggtttt**CCACCA**tcgcacgccggga**CCA**tcaccgtgtatgaagattcacaaccggggacgctg

**References**

1. Perez-Pinera, P., Kocak, D.D., Vockley, C.M., Adler, A.F., Kabadi, A.M., Polstein, L.R., Thakore, P.I., Glass, K.A., Ousterout, D.G. and Leong, K.W. (2013) RNA-guided gene activation by CRISPR-Cas9-based transcription factors. *Nature methods*, **10**, 973-976.

2. Sambrook, J., Fritsch, E.F. and Maniatis, T. (1989) *Molecular cloning*. Cold spring harbor laboratory press New York.

3. Howarth, M., Chinnapen, D.J.F., Gerrow, K., Dorrestein, P.C., Grandy, M.R., Kelleher, N.L., El-Husseini, A. and Ting, A.Y. (2006) A monovalent streptavidin with a single femtomolar biotin binding site. *Nature methods*, **3**, 267-273.

4. Wiggins, P.A., Van Der Heijden, T., Moreno-Herrero, F., Spakowitz, A., Phillips, R., Widom, J., Dekker, C. and Nelson, P.C. (2006) High flexibility of DNA on short length scales probed by atomic force microscopy. *Nature nanotechnology*, **1**, 137-141.

5. Gillespie, D.T. (1976) A general method for numerically simulating the stochastic time evolution of coupled chemical reactions. *Journal of computational physics*, **22**, 403-434.

6. SantaLucia, J., Allawi, H.T. and Seneviratne, P.A. (1996) Improved nearest-neighbor parameters for predicting DNA duplex stability. *Biochemistry*, **35**, 3555-3562.

7. Sugimoto, N., Nakano, S.-i., Katoh, M., Matsumura, A., Nakamuta, H., Ohmichi, T., Yoneyama, M. and Sasaki, M. (1995) Thermodynamic parameters to predict stability of RNA/DNA hybrid duplexes. *Biochemistry*, **34**, 11211-11216.

8. Watkins, N.E., Jr., Kennelly, W.J., Tsay, M.J., Tuin, A., Swenson, L., Lee, H.R., Morosyuk, S., Hicks, D.A. and Santalucia, J., Jr. (2011) Thermodynamic contributions of single internal rA.dA, rC.dC, rG.dG and rU.dT mismatches in RNA/DNA duplexes. *Nucleic acids research*, **39**, 1894-1902.

9. Hsu, P.D., Scott, D.A., Weinstein, J.A., Ran, F.A., Konermann, S., Agarwala, V., Li, Y., Fine, E.J., Wu, X. and Shalem, O. (2013) DNA targeting specificity of RNA-guided Cas9 nucleases. *Nature biotechnology*, **31**, 827-832.

10. Ratcliff, G.C. and Erie, D.A. (2001) A novel single-molecule study to determine protein-protein association constants. *Journal of the American Chemical Society*, **123**, 5632-5635.

11. Yang, Y., Sass, L.E., Du, C., Hsieh, P. and Erie, D.A. (2005) Determination of protein-DNA binding constants and specificities from statistical analyses of single molecules: MutS-DNA interactions. *Nucleic acids research*, **33**, 4322-4334.

12. Rodriguez, A. and Laio, A. (2014) Machine learning. Clustering by fast search and find of density peaks. *Science (New York, N.Y.)*, **344**, 1492-1496.

13. Sakmann, B. and Neher, E. (1995) Single-channel recording.

14. Sternberg, S.H., Redding, S., Jinek, M., Greene, E.C. and Doudna, J.A. (2014) DNA interrogation by the CRISPR RNA-guided endonuclease Cas9. *Nature*.
